# Developing a Core Outcome Set for the Evaluation of Remote Patient Monitoring Interventions Using the Sextuple Aim: Modified Delphi Study

**DOI:** 10.2196/92863

**Published:** 2026-07-15

**Authors:** Anna Heilig, Tobias Bonten, Kasper Recourt, M Elske van den Akker-van Marle

**Affiliations:** 1 Department of Medical Decision Making, Leiden University Medical Center, Albinusdreef 2, Leiden, South Holland, 2333 ZA, The Netherlands, 31 0630205719; 2 Department of Public Health and Primary Care, Leiden University Medical Center, Leiden, The Netherlands; 3 National eHealth Living Lab (NeLL), Leiden University Medical Center, Leiden, The Netherlands

**Keywords:** digital health, remote patient monitoring, value assessment framework, Sextuple Aim, core outcome set, Delphi, telemonitoring, remote monitoring

## Abstract

**Background:**

The rapid expansion of remote patient monitoring (RPM) interventions highlights the need for their comparison and evaluation. Current evaluation frameworks often fail to capture a multistakeholder perspective. Traditional health technology assessment approaches emphasize health and economic outcomes, providing an incomplete picture of RPM’s broader impact. The Sextuple Aim, encompassing health outcomes, costs, patient and provider experience, equity, and sustainability, offers a more comprehensive approach. Yet, the specific aspects that are important for capturing each domain for diverse stakeholders, and that could form a core outcome set (COS) for RPM evaluation, remain undefined.

**Objective:**

This study aimed to identify the most important value aspects for evaluating RPM interventions from the Sextuple Aim domains.

**Methods:**

Value aspects relevant to evaluating RPM interventions within the Sextuple Aim framework were identified in a review of the literature, and this initial set was refined through stakeholder meetings. Subsequently, 6 stakeholder groups completed 3 Delphi rounds. Patients and informal caregivers (25‐30 respondents per group) were recruited via a patient federation. Health care providers (20‐30 experts), managers, insurers, and researchers (10‐20 experts per group) were recruited. In each round, participants rated the importance of aspects on a 5-point Likert scale. In round 2, participants ranked aspects within each domain to determine their relative importance; in round 3, they ranked aspects across domains. A multicriteria consensus rule was used to select aspects for the preliminary COS. The COS was finalized during a multistakeholder expert meeting with 7 experts.

**Results:**

Of the 171 respondents, 137 (80%) completed all Delphi rounds. The input set, formed through a literature search and meetings, contained 43 value aspects. The total set contained 47 aspects after the first round. After all 3 rating rounds, 33 of 47 (70%) value aspects met the predefined importance threshold. Ranking results from rounds 2 and 3 identified aspects prioritized within and across domains. In total, 26 (55%) aspects met the multicriteria consensus rule and formed the preliminary COS. During the final multistakeholder expert meeting involving subgroup exercises and moderated discussions, value aspects with conceptual overlap were merged, removed, or relocated. The final COS included 17 value aspects, with 1 additional emergent aspect identified during the expert meeting and included as a recommended item, which also resulted in full Sextuple Aim domain coverage.

**Conclusions:**

An RPM Sextuple Aim COS comprising 17 value aspects and 1 recommended aspect was developed, providing a multidimensional and multistakeholder evaluation set. This COS provides a standardized, comprehensive basis for evaluating RPM interventions and can be used to support the validation and refinement of existing RPM evaluation tools. Future research may consider operationalizing the COS and its recommended aspect into measurable items and assessing its feasibility and uptake in practice.

## Introduction

The application of digital health technologies (DHTs) was expedited by the COVID-19 pandemic, and, to date, health care systems have become increasingly reliant on them [1,2]. The integration of DHTs into health care has highlighted their potential to offer solutions to the significant pressures under which health care systems worldwide are operating [3,4]. Consequently, there is an increasing need to generate evidence to determine which interventions are the most beneficial and should be widely implemented, which is the last step in the eHealth evaluation cycle [5,6]. One widely adopted instrument across countries for this is health technology assessment (HTA), which was originally developed to evaluate the effectiveness and economic value of nondigital health interventions (eg, pharmaceuticals) [7]. This instrument is also frequently used to inform decision-making for DHTs [8,9]. However, the health-related quality of life (QoL) and cost measures it includes do not completely capture the value of DHTs (including remote patient monitoring [RPM]) for different stakeholders, such as patients and health care providers [10,11].

The Quadruple Aim, which encompasses 4 health care goals (improved population health, improved patient and provider experience, and reduced costs), captures a broader value compared to HTA [12]. However, the existing literature does not examine all of these domains equally [13,14]. Additionally, the Quadruple Aim has expanded to include equity [15], a domain that is also highly relevant for DHTs [16]. A review emphasized the need for validated tools to quantitatively assess the impact of DHTs on equity [17]. This framework was further extended to the Sextuple Aim by incorporating sustainability, reflecting increasing concerns about the environmental impact of health care on population health [18]. Comprehensive evaluation across these domains alone is insufficient; it is also important to consider the needs of key stakeholders (eg, health care providers, managers, insurers) when evaluating DHTs because their needs and definitions of health care value differ [19]. Two recent Delphi studies incorporated the knowledge and perspectives of different stakeholders to develop a set of indicators for evaluating digital or virtual health technologies in a health care setting [2,20].

DHTs encompass a wide range of applications, including electronic health records, mobile health, telehealth, and RPM. RPM is a key driver of the rapid expansion of DHTs and uses information technologies to enable remote surveillance of patients by facilitating the transmission of clinical data between patients and health care providers [21,22]. Moreover, RPM is distinguishable from other DHTs in that it involves active patient monitoring rather than passive monitoring and asynchronous patient-provider interaction [23,24]. Existing evaluation frameworks are more generic for DHTs or virtual health and may insufficiently capture these distinctive characteristics. Therefore, this study aims to identify the value aspects considered most important for assessing RPM interventions and to develop a multistakeholder core outcome set (COS) for RPM based on the Sextuple Aim. A modified Delphi design was chosen to iteratively build consensus among stakeholder groups, ensuring anonymity and independent judgments [25].

## Methods

### Overview

**Figure 1.**
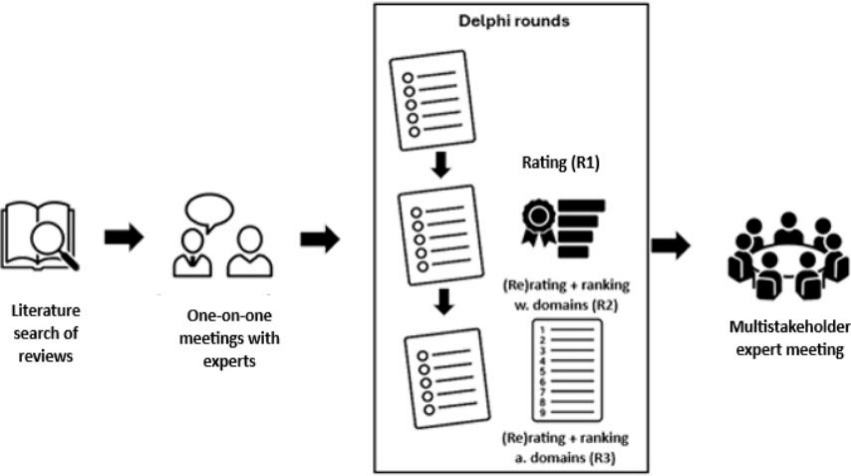
Schematic overview of the research process. R1: Round 1; R2: Round 2; R3: Round 3; ranking w. domains: ranking within Sextuple Aim domains; ranking a. domains: ranking across Sextuple Aim domains.

To develop a COS, a combination of a literature search, one-on-one stakeholder meetings, 3 Delphi rounds, and a final expert meeting was conducted (Figure 1). This design was guided by the COMET (Core Outcome Measures in Effectiveness Trials) initiative, recommending outcome identification (via literature searches), prioritization (eg, Delphi-based methods), and consensus meetings [25]. Prior to the Delphi study, a literature search was conducted on reviews evaluating RPM interventions using (value) aspects of the Sextuple Aim (health, costs, patient experience, provider experience, equity, and sustainability) published in the last 10 years from 2016 to 2025 (Multimedia Appendix 1). Titles and abstracts of the reviews were independently screened by 2 reviewers and were eligible if they addressed 1 or more domains of the Sextuple Aim in the context of digital health. The full texts were then assessed, and potentially relevant value aspects were independently extracted by 2 researchers. Extracted aspects were discussed and consolidated to reach consensus. The preliminary list was further refined during meetings with experts, including patient representatives, health care providers, managers, insurers, and researchers. These experts were recruited through the researchers’ network. This process resulted in an initial list of 43 value aspects. More details on this process, along with an overview and their definitions, are presented in Multimedia Appendix 2. These value aspects and their descriptions were incorporated into the Castor EDC online questionnaire tool for the first Delphi round (R1). For the second (R2) and third (R3) rounds, the online survey software tool Alchemer was used in order to conduct the ranking exercises. The findings of this study were reported in accordance with the ACCORD (Accurate Consensus Reporting Document) checklist (Checklist 1) [26].

### Recruitment and Eligibility

Participants from 6 stakeholder groups involved in using or evaluating an RPM application in the care pathway were included: patients, informal caregivers or household members, health care providers, managers, insurers, and researchers. Recruitment occurred from March to April 2025. Respondents had to be fluent in Dutch and have experience with digital health applications. Patients and informal caregivers were recruited via the Dutch Patient Federation (Ikzoekeenpatiënt) [27]. The federation disseminated an invitation via its online platform, including a study description, eligibility criteria, and compensation for participation, and individuals could sign up. The federation ensured a diverse and representative sample of patients and informal caregivers with respect to age, sex, and health conditions. The other groups were recruited using snowball sampling, where initial participants from the researchers’ network were asked to participate and share the study invitation within their own networks. Recruitment channels included (1) online flyers distributed through stakeholder networks and LinkedIn and (2) direct outreach through hospital contacts. We aimed for 25 to 30 patients and informal caregivers per group, 20 to 30 health care providers, and 10 to 20 insurers, managers, and researchers in each group. The target sample size was at least 2-digit numbers per group, which was sufficient to support meaningful consensus [28]. The maximum target was 30 participants, as it is known that additional participants offer limited benefit and may reduce response rates [29]. Sample sizes per group were further aligned to balance diversity and feasibility.

### Pre-Delphi Steps

Before each Delphi round, the online questionnaire was piloted with laypeople to assess its clarity, perceived length, and ease of completion. The questionnaires were written in plain Dutch (Common European Framework of Reference for Languages B1), and value aspects were simplified (see Multimedia Appendix 2 for the original and simplified definitions) to ensure accessibility for all groups [30].

### Delphi Procedure

Each Delphi round began with a project description. In R1, respondents were asked to rate the importance of each value aspect for evaluating the value of RPM interventions on a 5-point Likert scale, ranging from “unimportant” to “very important.” Respondents were encouraged to provide a rationale and comment on each aspect and report missing aspects. A “no opinion” option was included for each aspect, since not all aspects might be relevant to all stakeholder groups.

In R2, respondents first rerated the value aspects without consensus. They were provided with feedback (a graph showing the mean and frequency of the overall group and their stakeholder group, along with motivations). Respondents were also asked to rate the additional value aspects suggested in R1. Then, respondents ranked the value aspects within each domain from “most” (score=7 or a score equal to the number of value aspects within that domain) to “least” (score=1) important. For domains with more than 7 value aspects, respondents first selected the 7 they considered most important and then ranked them (unselected value aspects were assigned a score of 0). The items presented in the ranking exercise were randomized per domain for each respondent.

In R3, respondents rerated the value aspects without consensus and received comparable feedback as in R2. Then, respondents selected and ranked the 10 most important aspects across all domains from most (score=10) to least (score=1) important (unselected value aspects were assigned a score of 0). Again, the order of items was randomized.

To encourage timely questionnaire completion and increase response rates, reminders were sent out twice for each round (every 2 weeks), except for insurers, who received a third reminder for R2 [31]. To reduce group pressure and conformity bias, anonymity was ensured [32,33].

### Data Analysis and Selection Criteria

The anonymized data were analyzed using SPSS software (version 29; IBM Corp). The results of the Delphi ratings were analyzed by calculating percentages and IQRs. To analyze the overall group rating results, these metrics were weighted to account for different group sizes. The IQR represented the distance between the 25th and 75th percentile values in the rating (a smaller IQR represented greater consensus). Consensus on importance was reached when at least 75% of respondents (excluding those with “no opinion”) rated a value aspect as “important” or “very important,” and the IQR was ≤1. Both metrics were used to ensure high agreement and limited dispersion [34]. Similarly, consensus of unimportance was defined as at least 75% of participants rating a value aspect as “unimportant” or “somewhat important” together with an IQR ≤1. If no consensus was reached, the value aspect was rerated in the subsequent round. To analyze the ranking results of value aspects within domains, mean rank scores were normalized to a common scale ranging from 0 to 100 for each stakeholder group (Multimedia Appendix 3). This normalization accounted for differences in the number of value aspects per domain, ensuring comparability across domains. Higher normalized ranking scores indicated a higher relative priority within that domain. To calculate the overall ranking within the domains, the normalized scores were weighted by stakeholder group size. Complete-case sensitivity analyses assessed the impact of respondent retention within and between groups on the rated importance and ranking within domains of value aspects. The ratings and rankings within domains for respondents who completed all 3 rounds were analyzed similarly to the full sample, and the results were compared.

In the overall ranking across domains, respondents selected and then ranked the 10 value aspects. Mean ranks were calculated for each group, as well as an overall weighted mean. In a sensitivity analysis, the unselected aspects were assigned a negative score of −10 to replace the assumption of neutrality with active deprioritization. This value was chosen to generate the largest possible separation from the selected and ranked aspects (scored 1‐10) while remaining within the ranking ranges.

A multicriteria consensus rule was used to select value aspects for a preliminary COS. These criteria were derived from the literature and input from experts [20,35]. This a priori defined rule combined rating and ranking results to integrate absolute importance and relative prioritization. Value aspects were included in the COS if they met 2 of the following criteria: (1) rated as “important” or “very important” by ≥75% of respondents and IQR ≤1 (the importance-consensus criterion); (2) the mean ranking was higher than or equal to that in a hypothetical situation in which all value aspects within the domain were considered equally important (the ranking within domains criterion); and (3) the weighted mean of the overall ranking was in the top 25 (the ranking across domains criterion).

### Expert Meeting

Following the Delphi rounds, consensus on the COS was reached at a 3-hour expert meeting held in September 2025. We aimed to recruit 10 experts, purposively sampled to ensure representation from various stakeholder groups while maintaining a manageable group size that would facilitate in-depth discussion and promote equal contributions. Due to scheduling constraints, 7 experts participated: 3 researchers, 2 managers, 1 provider, and 1 patient or informal caregiver representative from the Dutch Patient Federation. Three of the 7 experts had also participated in the Delphi study, providing continuity with the previous rounds while also ensuring independent perspectives, thereby reducing overreliance on prior outcomes. Despite multiple recruitment attempts, no insurer was able to attend this meeting; therefore, the insurer perspective was only represented in the Delphi results. During the meeting, 2 subgroups were formed to ensure balanced representation within each one. One subgroup consisted of 2 researchers (1 of whom had clinical experience), a manager, and a patient representative, while the other subgroup comprised a researcher, a health care provider, and a manager. The experts first received written and verbal summaries of the rating and ranking results. The subgroups were then tasked with proposing a COS based on the preliminary COS derived from the predefined multicriteria consensus rule. Discussions were guided by the results of the Delphi rounds, and any discrepancies were addressed in a moderated discussion. The moderator, a member of the research team with expertise in evaluating interventions and developing outcome measures, ensured equal participation and had no direct interest in the outcomes. To minimize moderator-induced bias, the moderator’s role was limited to facilitating discussion and summarizing findings, in accordance with the COMET guidelines [25]. Proposed changes to the COS were discussed until a unanimous decision was reached. Through this iterative, moderated process, a final COS was agreed upon.

### Ethical Considerations

The Leiden University Medical Center’s non–*Wet Medisch-Wetenschappelijk Onderzoek* committee approved the study. The committee deemed the study not subject to the Dutch Medical Research Involving Human Subjects Act (nWMO-D4-2024-018). All participants received written information about the study’s objectives and anticipated benefits. For patients and informal caregivers, the information was written in plain Dutch (Common European Framework of Reference for Languages B1). All participants provided informed consent before enrolling in the study (Multimedia Appendix 4). Participation was voluntary, and participants could withdraw at any time without consequence. Patients and informal caregivers received a €25 gift (US $29.30, averaged exchange rate in September 2025) card after completing all 3 rounds. External experts participating in the expert meeting were reimbursed for their time and travel expenses but received no direct financial incentives for participation. Before analyzing the data, patient-identifying information and anonymized study data were separated. The patient-identifying information was saved on a separate disk, which was only accessible to the 2 main researchers.

## Results

### Delphi Participants

The demographics of the participants in the different stakeholder groups are described in Table 1. A total of 171 participants completed R1, of whom 148 (87%) completed R2 and 137 (80%) completed R3. Retention rates varied among the groups, ranging from 97% for patients to 50% for insurers.

**Table 1.** Characteristics of participants in the Delphi study.

Participants	Participants in R1^a^ (N=171), n (%)	Participants in R2^b^, n (%)	Participants in R3^c^, n (%)	Age in R1 (y), mean (SD)	Women in R1, n (%)
Patients	58 (33.9)	56 (97)	56 (97)	63.4 (11.1)	32 (55)
Informal caregivers	45 (26.3)	40 (89)	37 (82)	65.0 (12.1)	29 (64)
Health care providers	27 (15.8)	18 (67)	15 (56)	42.5 (10.3)	17 (63)
Health care managers	23 (13.5)	19 (83)	17 (74)	44.7 (11)	17 (74)
Health care insurers	8 (4.7)	5 (63)	4 (50)	44.9 (13)	3 (38)
Researchers	10 (5.8)	10 (100)	9 (90)	31.9 (4.9)	7 (70)

a R1: round 1.

b R2: round 2.

c R3: round 3.

### Results of Weighted Rating

**Figure 2.**
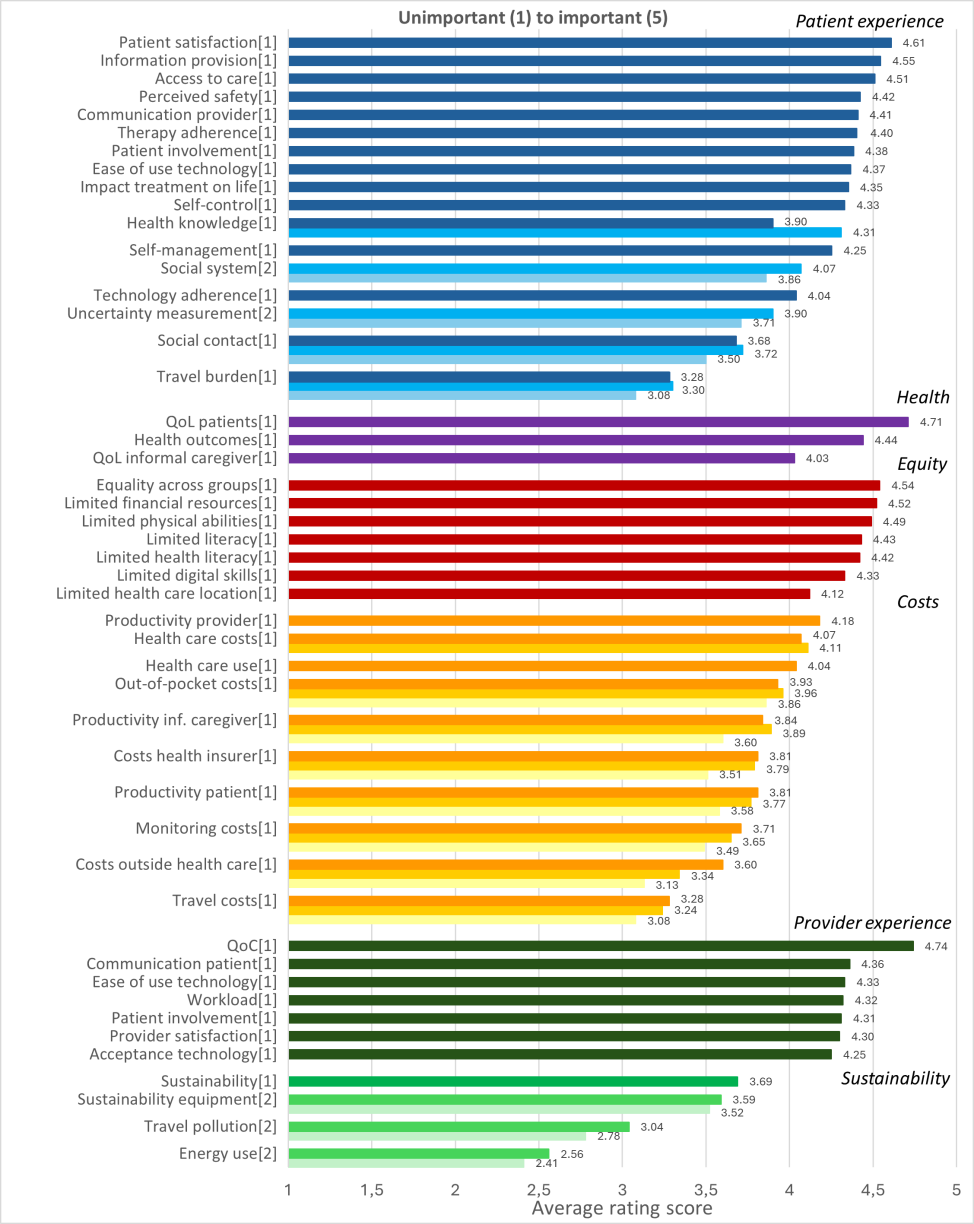
All group mean weighted rating of value aspects in the 3 rounds of the Delphi study; bar shading denotes the rating round: lighter bars represent aspects rerated in later rounds (R2 or R3) due to lack of prior consensus or because they were newly introduced in R2. [X] indicates the round in which an aspect was introduced. inf.: informal; QoC: quality of care; QoL: quality of life.

The rating of value aspects was conducted in all 3 rounds. In R2 and R3, aspects for which no consensus had been reached were rerated. In R1, respondents rated the initial set of 43 value aspects derived from the literature review and meetings with experts (Figure 2). In R1, for 31 value aspects, consensus on importance was met, as the rated importance was ≥75% and the IQR was ≤1 (Table 2). None of the aspects met the unimportance-consensus criterion. All aspects within the health, equity, and provider experience domains met the importance-consensus criterion. Twelve value aspects did not meet consensus on either criterion for importance or unimportance and, hence, had to be rerated in the subsequent round: 3 from the patient experience domain, the sustainability value aspect, and 8 from the cost domain. Managers were the only stakeholder group for which “sustainability” met the importance-consensus criterion. See Multimedia Appendix 5 for the distribution of the value aspects lacking consensus across groups.

**Table 2.** Heatmap of final percentage perceived importance (“important” or “very important”) and IQR.

Value aspect [final round rated]^a^	Final percentage perceived importance (IQR)^b^						
	Overall^c^	Patient	Caregiver	Provider	Manager	Insurer	Researcher
Patient experience							
Patient satisfaction [1]	94.6 (1.0)^d^	98.3 (1.0)^d^	97.8 (1.0)^d^	92.6 (1.0)^d^	91.3 (1.0)^d^	87.5 (1.0)^d^	100.0 (1.0)^d^
Access to care [1]	93.4 (1.0)^d^	98.3 (1.0)^d^	100 (1.0)^d^	88.8 (1.0)^d^	95.6 (1.0)^d^	87.5 (1.0)^d^	90.0 (1.0)^d^
Information provision [1]	92.6 (1.0)^d^	98.3 (0.0)^d^	100 (1.0)^d^	100.0 (1.0)^d^	100.0 (0.0)^d^	87.5 (1.0)^d^	70.0 (2.0)^e^
Therapy adherence [1]	92.0 (1.0)^d^	100 (1.0)^d^	93.3 (1.0)^d^	100.0 (1.0)^d^	91.3 (1.0)^d^	87.5 (1.0)^d^	80.0 (1.3)^f^
Communication provider [1]	91.8 (1.0)^d^	94.8 (1.0)^d^	100.0 (0.5)^d^	92.6 (1.0)^d^	95.6 (1.0)^d^	87.5 (1.0)^d^	80.0 (0.5)^f^
Patient involvement [1]	90.2 (1.0)^d^	94.8 (1.0)^d^	100.0 (1.0)^d^	88.8 (1.0)^d^	100.0 (1.0)^d^	87.5 (1.0)^d^	70.0 (1.3)^e^
Perceived safety [1]	89.5 (1.0)^d^	93.1 (1.0)^d^	97.7 (1.0)^d^	92.6 (1.0)^d^	91.3 (1.0)^d^	62.5 (2.0)^e^	100.0 (1.0)^d^
Ease of use technology [1]	88.7 (1.0)^d^	87.8 (1.0)^d^	91.1 (1.0)^d^	92.5 (1.0)^d^	95.6 (1.0)^d^	75.0 (1.8)^f^	90.0 (1.0)^d^
Impact treatment [1]	88.4 (1.0)^d^	93.1 (1.0)^d^	84.4 (1.0)^f^	100.0 (1.0)^d^	100.0 (1.0)^d^	62.5 (2.0)^e^	90.0 (1.0)^d^
Self-control [1]	85.8 (1.0)^f^	79.3 (1.0)^f^	95.6 (1.0)^d^	85.1 (1.0)^f^	85.1 (1.0)^f^	75.0 (1.8)^f^	80.0 (1.3)^f^
Health knowledge [2]	84.6 (1.0)^f^	92.6 (1.0)^d^	87.5 (1.0)^d^	100.0 (1.0)^d^	89.5 (1.0)^d^	60.0 (1.0)^g^	77.8 (0.2)^f^
Self-management [1]	83.4 (1.0)^f^	87.5 (1.0)^d^	90.9 (1.0)^d^	88.9 (1.0)^d^	95.6 (1.0)^d^	87.5 (1.0)^d^	50.0 (1.3)^g^
Technology adherence [1]	77.5 (1.0)^f^	91.0 (1.0)^d^	83.7 (1.0)^f^	70.4 (1.0)^e^	82.6 (1.0)^f^	87.5 (1.0)^d^	50.0 (1.0)^g^
Uncertainty measurements [3]	73.4 (1.0)^e^	48.2 (2.0)^h^	61.1 (1.0)^g^	66.7 (1.0)^e^	70.6 (1.5)^e^	100.0 (0.0)^d^	100.0 (0.0)^d^
Social system [3]	73.0 (2.0)^e^	72.7 (2.0)^e^	77.1 (1.0)^f^	80.0 (1.0)^f^	70.5 (1.0)^e^	75.0 (1.5)^f^	62.5 (1.0)^e^
Social contact [3]	58.7 (2.0)^g^	71.5 (2.0)^e^	67.6 (1.0)^e^	60.0 (2.0)^g^	53.0 (1.0)^g^	50.0 (2.0)^g^	50.0 (1.8)^g^
Travel burden [3]	35.3 (2.0)^i^	48.2 (2.0)^h^	58.4 (2.0)^g^	33.4 (2.0)^i^	47.1 (1.0)^h^	0.0 (0.8)^j^	25.0 (1.8)^i^
Health							
QoL^k^ patients [1]	97.7 (1.0)^d^	98.2 (0.0)^d^	97.8 (0.0)^d^	100.0 (0.0)^d^	100.0 (1.0)^d^	100.0 (0.8)^d^	90.0 (1.0)^d^
Health outcomes [1]	93.3 (1.0)^d^	94.6 (1.0)^d^	95.6 (1.0)^d^	92.6 (1.0)^d^	86.9 (1.0)^f^	100.0 (0.8)^d^	90.0 (1.0)^d^
QoL informal caregiver [1]	78.5 (1.0)^f^	92.0 (1.0)^d^	95.5 (1.0)^d^	88.9 (0.0)^d^	76.2 (0.5)^f^	62.5 (3.0)^e^	55.5 (1.0)^g^
Equity							
Equality across groups [1]	95.4 (1.0)^d^	92.8 (1.0)^d^	93.3 (1.0)^d^	96.2 (1.0)^d^	100.0 (0.5)^d^	100.0 (0.8)^d^	90.0 (1.0)^d^
Limited physical abilities [1]	95.3 (1.0)^d^	96.5 (0.0)^d^	97.8 (1.0)^d^	100 (1.0)^d^	100 (1.0)^d^	87.5 (1.0)^d^	90.0 (1.0)^d^
Limited financial resources [1]	92.5 (1.0)^d^	89.4 (0.5)^d^	93.4 (1.0)^d^	96.3 (1.0)^d^	95.5 (0.3)^d^	100 (1.0)^d^	80.0 (1.3)^f^
Limited literacy [1]	91.5 (1.0)^d^	94.7 (1.0)^d^	91.1 (1.0)^d^	100.0 (1.0)^d^	95.7 (1.0)^d^	87.5 (1.0)^d^	80.0 (1.3)^f^
Limited health literacy [1]	90.7 (1.0)^d^	89.5 (1.0)^d^	90.9 (1.0)^d^	92.6 (1.0)^d^	91.3 (1.0)^d^	100.0 (1.0)^d^	80.0 (1.3)^f^
Limited digital skills [1]	89.8 (1.0)^d^	86.2 (1.0)^f^	86.4 (1.0)^f^	92.6 (1.0)^d^	95.6 (1.0)^d^	87.5 (1.0)^d^	90.0 (1.0)^d^
Limited health care location [1]	79.5 (1.0)^f^	93.1 (1.0)^d^	86.7 (1.0)^f^	80.0 (1.0)^f^	77.3 (1.3)^f^	50.0 (2.5)^g^	90.0 (1.0)^d^
Costs							
Health care costs [2]	86.1 (1.0)^f^	61.1 (2.0)^g^	77.5 (0.2)^f^	88.9 (1.0)^d^	100.0 (1.0)^d^	100.0 (1.0)^d^	88.9 (1.0)^d^
Productivity provider [1]	82.5 (1.0)^f^	80.8 (1.0)^f^	76.8 (1.0)^f^	81.4 (1.0)^f^	86.0 (1.0)^f^	100.0 (1.0)^d^	66.7 (1.0)^e^
Health care use [1]	76.6 (1.0)^f^	87.2 (1.0)^f^	90.7 (1.0)^d^	73.0 (1.0)^e^	77.2 (0.5)^f^	75.0 (1.8)^f^	55.5 (1.0)^g^
Out-of-pocket costs [3]	69.5 (2.0)^e^	68.5 (2.0)^e^	81.0 (1.0)^f^	53.3 (1.0)^g^	76.4 (1.0)^f^	50.0 (1.8)^g^	87.5 (0.8)^d^
Costs health insurer [3]	59.7 (1.0)^g^	46.5 (1.8)^h^	54.0 (1.5)^g^	46.7 (1.0)^h^	87.5 (0.0)^d^	100.0 (0.8)^d^	25.0 (1.5)^i^
Productivity informal caregiver [3]	59.4 (1.0)^g^	54.9 (1.0)^g^	75.6 (1.5)^f^	80.0 (1.0)^f^	70.6 (1.0)^e^	25.0 (1.8)^i^	50.0 (1.0)^g^
Monitoring costs [3]	58.7 (1.0)^g^	29.7 (2.0)^i^	54.0 (1.0)^g^	46.7 (1.0)^h^	70.6 (1.0)^e^	75.0 (1.5)^f^	75.0 (0.8)^f^
Productivity patient [3]	56.8 (1.0)^g^	40.4 (2.0)^h^	62.1 (1.0)^g^	86.7 (1.0)^f^	88.2 (1.0)^d^	0.0 (0.8)^j^	62.5 (1.0)^e^
Travel costs [3]	40.5 (2.0)^h^	50.0 (2.8)^g^	54.0 (1.0)^g^	60.0 (1.0)^g^	41.2 (2.0)^h^	0.0 (1.5)^j^	37.5 (2.0)^h^
Costs outside of health care [3]	39.6 (2.0)^h^	45.5 (2.0)^h^	42.9 (1.0)^h^	46.7 (1.0)^h^	35.3 (2.0)^i^	25.0 (1.8)^i^	42.9 (2.0)^h^
Provider experience							
QoC^l^ [1]	98.7 (0.0)^d^	98.2 (1.0)^d^	97.7 (0.0)^d^	96.3 (0.0)^d^	100.0 (0.0)^d^	100.0 (1.0)^d^	100 (1.0)^d^
Patient involvement [1]	89.9 (1.0)^d^	98.3 (1.0)^d^	95.5 (1.0)^d^	88.9 (1.0)^d^	86.9 (1.0)^f^	100.0 (1.0)^d^	70.0 (1.3)^e^
Workload [1]	87.8 (1.0)^d^	86.7 (1.0)^f^	81.8 (1.0)^f^	88.8 (1.0)^d^	95.6 (1.0)^d^	75.0 (1.5)^f^	100.0 (1.0)^d^
Provider satisfaction [1]	86.3 (1.0)^f^	85.4 (1.0)^f^	84.1 (1.0)^f^	88.8 (1.0)^d^	95.6 (1.0)^d^	75.0 (1.8)^f^	88.9 (1.0)^d^
Ease of use technology [1]	86.0 (1.0)^f^	91.1 (1.0)^d^	88.7 (1.0)^d^	88.9 (1.0)^d^	82.6 (1.0)^f^	75.0 (1.8)^f^	90.0 (1.0)^d^
Communication patient [1]	85.7 (1.0)^f^	98.3 (1.0)^d^	93.2 (0.0)^d^	88.8 (1.0)^d^	91.3 (1.0)^d^	71.5 (2.0)^e^	70.0 (2.0)^e^
Acceptance technology [1]	85.3 (1.0)^f^	85.7 (1.0)^f^	81.4 (1.0)^f^	81.4 (1.0)^f^	95.6 (1.0)^d^	87.5 (1.0)^d^	80.0 (1.3)^f^
Sustainability							
Sustainability [1]	60.2 (1.3)^g^	51.0 (2.0)^g^	57.8 (1.5)^g^	68.1 (1.0)^e^	79.1 (0.3)^f^	45.0 (2.0)^h^	33.3 (2.0)^i^
Reusability equipment [3]	57.0 (1.0)^g^	54.6 (3.0)^g^	56.7 (2.0)^g^	53.3 (1.0)^g^	64.7 (1.0)^e^	50.0 (1.8)^g^	62.5 (1.8)^e^
Pollution travel [3]	30.8 (2.0)^i^	38.1 (2.0)^h^	40.5 (2.0)^h^	40.0 (2.0)^h^	41.2 (1.5)^h^	0.0 (0.8)^j^	25.0 (1.8)^i^
Energy use [3]	13.7 (1.0)^m^	29.1 (3.0)^i^	22.2 (1.0)^m^	6.7 (1.0)^j^	11.8 (1.0)^j^	0.0 (0.0)^j^	12.5 (1.0)^m^

a [Final round rated]: the last round in which a value aspect was rated; when consensus was reached, it was not rerated in the subsequent round.

b Final percentage perceived importance (IQR): the percentage perceived importance and IQR for an aspect as rated in the last round an aspect was (re)rated.

c Results were weighted to correct for differences in sample size.

d Sample size: ≥87.5 to 100.

e Sample size: ≥62.5 to <75.0.

f Sample size: ≥75.0 to <87.5.

g Sample size: ≥50.0 to <62.5.

h Sample size: ≥37.5 to <50.0.

i Sample size: ≥25.0 to <37.5.

j Sample size: ≥0.0 to <12.5.

k QoL: quality of life.

l QoC: quality of care.

m Sample size: ≥12.5 to <25.0.

Respondents’ suggestions led to the addition of the aspects “social system” and “uncertainty from measurements” to the patient experience domain. Additionally, suggestions within the sustainability domain resulted in the value aspect “sustainability” being split into 3.

After R2, the aspects “health knowledge” and “health care costs” met the importance-consensus criterion. The remaining 14 aspects were rerated in R3. In R3, these aspects were perceived as less important than in R2 and did not meet the consensus criteria for importance or unimportance. Ultimately, 33 of 47 (70%) aspects met the importance-consensus criterion. Consensus was least often achieved in the sustainability domain, where all 3 value aspects (100%) fell below the threshold, followed by the cost domain (7 aspects, 70%), and, to a lesser extent, the patient experience domain (4 aspects, 24%). Divergence was observed between stakeholder groups regarding the importance of value aspects within the cost domain. Managers and the small group of insurers rated 6 and 5 value aspects as important, respectively, while the other stakeholder groups rated 1 (the researcher group) or at most 3 value aspects (the provider group) as important.

The “no opinion” option was infrequently selected across rounds, with patients using this option more often than other stakeholder groups (Multimedia Appendix 6).

### Results of Weighted Ranking Within Domains

**Figure 3.**
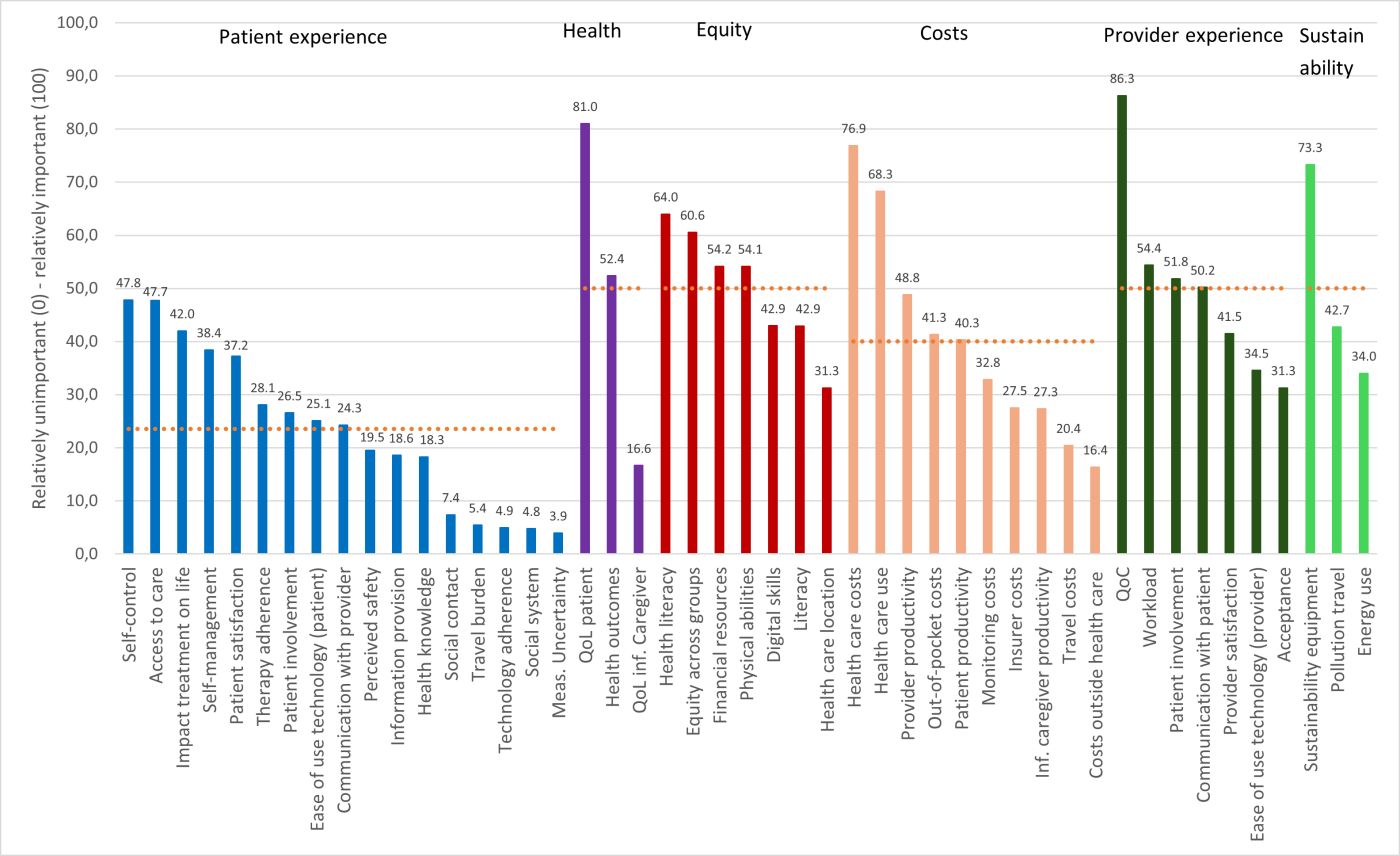
All groups normalized (weighted) mean rankings within domains; the orange line represents the average value if all value aspects had been ranked equally. inf.: informal; Meas.: measurement; QoC: quality of care; QoL: quality of life.

The ranking of value aspects within each domain reflects their relative prioritization (Figure 3). Across domains, approximately half of the value aspects were prioritized above the domain average, which provided differentiation in relative importance. Value aspects scoring above the domain average (ie, the expected score if all aspects were equally ranked) are above the reference line (Figure 3). According to this ranking, 25 of 47 (53%) aspects were ranked above average and, hence, met the ranking within domains criterion. See Multimedia Appendix 7 for the ranking of the aspects within domains by the individual stakeholder groups. No remarkable differences were found among the stakeholder groups.

### Results of Weighted Ranking Across Domains

The ranking across domains exercise examined the relative priority of value aspects across all domains. Overall, the across-domain ranking showed that value aspects within the health, provider experience, patient experience, and equity domains were most frequently prioritized, whereas aspects from the cost domain were less often selected among the highest-ranked aspects. Value aspects met the ranking across domains criterion if they were in the overall top 25. The top 25 value aspects across domains included all health aspects (3/3), the majority of provider experience aspects (6/7), more than half of patient experience aspects (10/17) and equity aspects (4/7), and a limited number of cost aspects (2/10; Multimedia Appendix 8). The 10 highest-ranked value aspects of each group were incorporated into the overall top 25, except for “out-of-pocket costs,” which was in the patients’ top 10 but not in the overall top 25. All stakeholder groups included “patient QoL” and “quality of care (QoC)” in their top 10. All groups except managers ranked “patient QoL” as number 1, whereas managers ranked it third and “QoC” as first.

The sensitivity analysis (deprioritization instead of neutrality) only minorly changed the top 25 value aspects included (Multimedia Appendix 9). Within the top 25, “limited financial resources” was removed from the top 25 (from 25th to 27th place), while “information provision” was added (from 26th to 24th place).

### Multicriteria Consensus Rule and Expert Meeting

Following the multicriteria consensus rule, the preliminary COS included 26 value aspects. Twenty-one (81%) aspects met all 3 criteria (Multimedia Appendix 10). All of the value aspects in the preliminary COS met the importance-consensus criterion. Of the 5 (19%) aspects meeting only 2 criteria, 4 did not meet the ranking within domains criterion, and 1 did not meet the ranking across domains criterion. The complete-case sensitivity analyses revealed no discrepancies in the value aspects that met the importance-consensus criterion and the within-domains ranking of value aspects (Multimedia Appendix 11), as the same aspects were retained in the preliminary COS.

During the expert meeting, the subgroups created a COS informed by the results of the ratings and rankings. The subgroup outputs were then discussed in a moderated plenary session, where value aspects were merged, removed, or relocated when consensus was reached. Value aspects were merged when they were considered to be conceptually overlapping and thus reflected the same underlying construct. This was identified among aspects within the patient experience, cost, and provider experience domains (Textbox 1). These were combined into 3 paired value aspects. For instance, the aspects “patient involvement” and “communication with patients” within the provider experience domain were merged, as the experts indicated that meaningful communication with patients is grounded in patient involvement. Value aspects were removed when they were judged to represent outcomes arising from other included aspects rather than standalone values. For instance, “patient satisfaction” was removed from the patient experience domain because it was viewed as the cumulative result of other patient experience aspects and may depend more on a patient’s momentary mood than on their experience with the RPM intervention [36]. Relocation occurred when an aspect aligned more closely with a different Sextuple Aim domain based on its meaning. “Access to care” was relocated from the patient experience domain and merged with 5 aspects from the equity domain and added to it because these aspects were considered fundamental to access. “Sustainability” did not meet the multicriteria consensus rule, but its emergent relevance for the long-term viability of health care systems was acknowledged during the expert meeting. Following this, sustainability was included as a recommended value aspect in the COS.

Textbox 1. **Core outcome set based on Delphi results and expert meeting.**

**Patient experience**

- Self-control
- Self-management
- Impact on treatment and life
- Therapy adherence
- Patient involvement
- Ease of use of technology
- Perceived safety
- Communication with provider

**Health**

- Quality of life, patient
- Health outcomes
- Quality of life, informal caregiver

**Provider experience**

- Experienced quality of care
- Workload
- Communication with patient

**Equity**

- Access to care (health literacy, financial resources, physical abilities, digital skills, and equality across groups)

**Costs**

- Health care costs
- Provider productivity

**Sustainability**

- Sustainability (included as a recommended value aspect rather than a required value aspect)

## Discussion

### Principal Findings

Applying the multicriteria consensus rule to the Delphi results and subsequent expert meeting yielded a final RPM Sextuple Aim COS comprising 17 value aspects and 1 recommended aspect. This COS extends beyond traditional HTA domains, incorporating value aspects from the cost and health domains as well as 12 aspects from the patient experience, provider experience, and equity domains. This emphasizes that the value of RPM interventions is reflected in broader dimensions. Notably, no value aspects within the sustainability domain met the multicriteria consensus rule. However, during the expert meeting, experts recommended including sustainability as a value aspect in the final COS, as there was consensus on its emergent relevance for the long-term viability of health care systems.

Notable differences were observed between stakeholder groups for value aspects within the cost domain. Managers and the small group of insurers consistently rated these value aspects as more important than other stakeholder groups. These findings are supported by a previous study, which observed that the importance of costs when evaluating an intervention depends on the stakeholder group, with costs also being considered more important by managers and insurers than by health care providers [37].

### Comparison With Prior Work

Two previous multistakeholder Delphi studies have established sets of items for evaluating DHTs and virtual care, using the Quadruple Aim and Quintuple Aim, respectively [2,20]. Our research contributes to current knowledge by specifically focusing on RPM, the largest contributor to the expansion of DHTs [21]. Petrie et al [20] identified similar items (within provider experiences: eg, QoC and workload) but also reported different items. In this study, we explicitly included aspects belonging to the effectiveness and uptake phases of the eHealth evaluation cycle (eg, communication with providers), whereas Petrie et al [20] also incorporated items related to earlier phases (eg, interoperability and portability) [6].

Although Haig et al [2] addressed the Quadruple Aim, they also included a sustainability item on which no consensus was reached. This finding aligns with the results of this study. Although studies show that environmental sustainability is increasingly viewed as important among stakeholder groups, it is still rarely incorporated into health care decision-making [38,39]. This was also observed in decisions regarding the adoption of telemonitoring [40]. One possible explanation for this discrepancy may be that the necessary expertise, metrics, and practical tools needed to account for environmental impacts are still emerging and have not yet been embedded in routine assessment and decision-making processes. The same holds for equity. A recent literature review revealed that equity is rarely assessed in RPM research, which underscores the need for a validated tool to quantitatively assess this dimension [17].

The interpretability, coherence, and comprehensiveness of the final COS were ensured at the expert meeting, where the findings of the Delphi rounds were discussed, and conceptually overlapping value aspects were merged [41].

### Limitations

This study has limitations. First, we aimed to include 10 to 20 insurers and researchers based on availability and feasibility. However, only 8 insurers participated in R1, meaning that the aim was not met, and the insurer perspective was underrepresented relative to the other stakeholder groups. This may have been offset by weighting the results by stakeholder group. Second, although the retention rate was comparable to or higher than that of previous digital-application Delphi studies [2,20], attrition was substantial in the insurer group, where only half of the initial participants completed R3. To assess the potential impact of this dropout, we conducted a sensitivity analysis including only respondents who completed all 3 rounds. These complete-case analyses showed no meaningful changes in the results of rating and within-domain ranking (Multimedia Appendix 11): the same value aspects were retained in the preliminary COS, suggesting that participants who discontinued had similar views to those who completed all rounds. Third, the composition of the expert meeting differed from that of the Delphi process, with a higher proportion of researchers present and no insurer representation. As this meeting was used to finalize the COS, the imbalance in stakeholder representation may have influenced the final prioritization decisions. Although discussions were moderated and structured to encourage equal participation, imbalances in the composition of expert groups and the absence of key stakeholder perspectives are recognized challenges in Delphi studies [28]. Fourth, the definitions of value aspects were simplified to ensure clarity and comprehensibility for all respondents. While this approach facilitated broader understanding and engagement, it may have slightly altered the scientific definition. For example, the definition of self-management focused on perceived control and ability to act, lacking the skill development aspect of the scientific definition [42]. Consequently, the importance attributed to certain value aspects might have differed if the scientific definition had been used. Even though simplified language was used in the Delphi rounds and the Dutch Patient Federation facilitated participant recruitment, including individuals with low literacy was not a goal of this study and remains a challenge [43]. Those with limited literacy may struggle with text-heavy questionnaires and abstract concepts, potentially introducing selection bias [43]. Selection bias could also arise from the snowball sampling recruitment approach used, which may have led to an overrepresentation of individuals more interested in or engaged with RPM. This could have influenced the prioritization of value aspects. Fifth, rating aspects is prone to agreement bias and nondifferentiation, with respondents tending to answer positively and show less variation [44]. The ratings indicate that 33 of 47 aspects would have been retained. To mitigate this tendency and encourage stronger discrimination between value aspects, we therefore also included ranking exercises. Lastly, this Delphi study was conducted within the Dutch health care context, which may limit the generalizability of the findings to other health care systems. Characteristics of the Dutch setting, such as short travel distances and its reimbursement structure, may have influenced the rating and ranking of value aspects, particularly those related to travel, access, and costs.

### Future Directions

The primary focus of this COS was to support HTA in improving existing evaluation tools used for telemonitoring, thereby supporting policy decision-making [45-47]. By systematically comparing existing tools with the COS, missing value aspects can be identified and incorporated, resulting in validated and comprehensive evaluation tools for assessing RPM interventions. This COS may be applied in broader terms, for example, by operationalizing its value aspects into measurable items to create an evaluation tool that can inform organizational and clinical decision-making, especially in the effectiveness and uptake phases of RPM development, where these aspects can be repeatedly assessed to capture evolving value. Future studies may focus on operationalizing and subsequently validating the COS. Another important direction for future research is the incorporation of perspectives from patients with limited (health) literacy. This inclusion would enhance the societal applicability of the COS, especially given concerns that the ongoing digital transformation may inadvertently increase the burden of health management on patients, potentially risking the widening of health disparities rather than promoting equity [42].

### Conclusions

This Delphi study constituted an RPM Sextuple Aim COS comprising 17 value aspects and 1 recommended value aspect, providing a multidimensional and multistakeholder evaluation set. This COS provides a standardized and comprehensive basis for evaluating and comparing RPM interventions and can be used to support the validation and refinement of existing RPM evaluation tools. Future research may consider operationalizing the COS and its recommended aspect into measurable items and assessing its feasibility and uptake in practice.

## Supplementary material

10.2196/92863 Multimedia Appendix 1 Search strategy.

10.2196/92863 Multimedia Appendix 2 Overview value aspects.

10.2196/92863 Multimedia Appendix 3 Calculation normalization ranking across domains.

10.2196/92863 Multimedia Appendix 4 Informed consent waiver.

10.2196/92863 Multimedia Appendix 5 Value aspects without consensus.

10.2196/92863 Multimedia Appendix 6 Attrition across rounds.

10.2196/92863 Multimedia Appendix 7 Table normalized mean rankings.

10.2196/92863 Multimedia Appendix 8 Table ranking across domains.

10.2196/92863 Multimedia Appendix 9 Sensitivity analysis deprioritization.

10.2196/92863 Multimedia Appendix 10 Preliminary core outcome set.

10.2196/92863 Multimedia Appendix 11 Complete-case sensitivity analyses.

10.2196/92863 Checklist 1 ACCORD checklist.

## Abbreviations

ACCORD: Accurate Consensus Reporting Document
COMET: Core Outcome Measures in Effectiveness Trials
COS: core outcome set
DHT: digital health tool
HTA: health technology assessment
QoC: quality of care
QoL: quality of life
RPM: remote patient monitoring
