# Supplementary material for: Developing a Core Outcome Set for the Evaluation of Remote Patient Monitoring Interventions Using the Sextuple Aim: Modified Delphi Study
Source: J Med Internet Res. 2026 Jul 15;28:e92863. doi: 10.2196/92863 (PMC13372298; doi:10.2196/92863)
Supplement: Multimedia Appendix 1 [file jmir-v28-e92863-s001.docx]

**Supplementary File 1 – Search strategy for input set of value aspects and one-on-one meetings with experts**

Applied search strategy

| PICO term | Key terms & alternatives |
| --- | --- |
| Patient, problem or population | N/A |
| Issue of interest or intervention | TITLE/ABSTRACT(“Digital Health” OR “Electronic Health” OR “ehealth” OR “Artificial Intelligence” OR “AI” OR “Diagnostic Software” OR “Remote Monitor*” OR Telemonitor*OR “Home Monitor*” OR Home-monitor* OR Telemetry OR “Remote Radiolog*” OR Teleradiolog* OR Telepatholog* OR “Remote Care” OR “Tele Rehabilitation*” OR “Remote Rehabilitation*” OR “Machine learning” OR “Online monitoring” OR “Digital Exclusion” OR “Technology Assessment, Biomedical”) OR Mesh(“Telemedicine”) |
| Comparison, control or comparator | N/A |
| Outcome | TITLE/ABSTRACT(“Quadruple aim” OR “Healthcare costs” OR “Medical Care Costs” OR “Treatment Costs” OR “Healthcare quality” OR “Quality of health care” OR “Quality of care” OR “Client Satisfaction” OR “Patients experiences” OR “Satisfaction” OR “Health professionals experience” OR “Experience of Health Care Professionals” OR “Health Professionals experiences” OR “Experience of Healthcare Professionals” OR “Healthcare professionals attitudes”) OR Mesh(“Healthcare costs” OR “Quality of health care” OR “Patient Satisfaction” OR “Patient Preference” OR “Attitude of Health Personnel”) |

One-on-one stakeholder meetings:

- Researchers from within the hospital (LUMC)
- Researchers from external hospitals
- Experts working at platform for evaluating digital health applications
- Insurer with experience in digital health reimbursement
- Patient representative (Patiëntenfederatie)
- Healthcare provider from within the hospital (LUMC)
- Healthcare manager from within the hospital (LUMC)
- Digital healthcare worker for the IT&D (implementer of digital healthcare concepts in the LUMC)
