# Supplementary material for: Developing a Core Outcome Set for the Evaluation of Remote Patient Monitoring Interventions Using the Sextuple Aim: Modified Delphi Study
Source: J Med Internet Res. 2026 Jul 15;28:e92863. doi: 10.2196/92863 (PMC13372298; doi:10.2196/92863)
Supplement: Multimedia Appendix 2 [file jmir-v28-e92863-s002.docx]

**Supplementary File 2 – Overview of value aspects included in the Delphi study and their definitions by domain**

| **Value aspect** | **Initial definition (in line with literature)** | **Definition used in Delphi study** |
| --- | --- | --- |
| ***Patient experience*** | | |
| Patient satisfaction | The extent to which a patient is satisfied with the healthcare they received from their healthcare provider or application. | The extent to which healthcare is delivered in a manner that aligns with the patient’s preferences and expectations. This includes satisfaction with healthcare providers, the treatment received, associated costs, and the time required to access and receive care. |
| Therapy adherence | The choice of patients to following through with the prescribed treatment while taking responsibility for their own well-being. | The extent to which a patient follows a healthcare provider’s recommendations and prescribed interventions. This may include, for example, taking medication as instructed or implementing lifestyle changes, such as smoking cessation, as advised by the healthcare provider. |
| Impact treatment on life | Illness induced disruptions to lifestyle activities, and interests that can compromise psychological well-being and contribute to emotional distress in disease. | The degree to which an intervention interferes with a patient’s daily life. This includes considerations such as the convenience of scheduling the treatment and the amount of time required for this each day. |
| Information provision | Information shared with patients to support patients’ understanding adjusted to the individual patient’s unique needs and capacity to process it. | The extent to which a patient receives sufficient and understandable information about their health status, treatment option, and use of digital tools. |
| Communication with provider | Mutual understanding between clinicians and patients regarding patient’s health needs, values, and perspectives and sharing power and responsibility. | The perceived quality of the interaction between the patient and the provider, both in person and through digital channels such as email, chat, or video consultations. It includes aspects such as whether the feels heard, feels comfortable asking questions, and if the patient perceives that sufficient time is devoted to the consultation. |
| Patient involvement | The inclusion of patient in decision making concerning their health, either on the individuals or the collective level. | The active participation of patients in health care decision-making, including shared decisions about treatment options, the use of digital tools, and the frequency of hospital visits. |
| Self-control | A process through which patients gain greater control over decisions and actions affecting their health. | The extent to which a patient has control over his/her treatment and feels a sense of autonomy in managing their care. This includes the ability to make personal decisions, such as when to take medication. |
| Social contact | A two-way conversation in the physical presence of another person. | The value a patient derives from interactions during the care process. This includes conversations with other patients in waiting rooms or with healthcare professionals, which can provide emotional support and reduce feelings of loneliness. |
| Health knowledge | The information people gather about health and illness that adds to their knowledge, meaning their beliefs about factors influencing health, causes of disease, and ways to treat and prevent illness increases. | A patient’s understanding of their health status, including the underlying causes of health problems and the expected impact of treatment on their overall health status. |
| Self-management | The practice of activities that individuals initiate and perform on their own behalf in maintaining life, health, and well-being and developing skills needed to devise, implement, evaluate, and revise an individualized plan for lifestyle change. | The extent to which a patient feels in control of his/her health and is able to take action when needed. It includes knowing what action to undertake to maintain well-being and to respond effectively to changes in health status. |
| Access to care | To opportunity to identify healthcare needs, to seek healthcare services, to reach, to obtain or use healthcare services and to actually have the need for services fulfilled. | The ease with which a patient can obtain the health care services he/she needs. This includes factors such as the ability to contact health care providers, the availability of services, waiting times, and the physical or logistical distance to care facilities. |
| Travel burden | Individual’s perception of barriers, such as the psychological, physical, and emotional strain associated with travelling for care. | The effort, time, cost, and stress a patient experiences when traveling to receive health care. This includes travel time, waiting time, transportation costs, and the physical or emotional burden associated with travel, such as fatigue or anxiety. |
| Ease of use of technology for the patient | A person’s perception of the usability of a particular system or technology when used within a defined context. | How easy and convenient it is for patients to interact with digital tools, such as apps or websites. This includes the usability, reliability, and technical performance of these tools to ensure they function properly without errors or interruptions. |
| Technology adherence | The degree to which individuals experience the content of the internet (digital) intervention. | The extent to which a patient uses digital tools as agreed with the healthcare provider. This includes, for example, consistent use of apps to report health measurements or other relevant data. |
| Perceived safety | Sense of security: an intrinsic state based on faith and trust in oneself and others/applications.  Feeling safe: perceived level of danger compared to the perceived level of comfort when interacting with applications. | Patient confidence in the healthcare system, including confidence that care is delivered correctly and without error, and that personal information is handled and stored securely. |
| ***Health*** | | |
| Quality of life patient | An individual’s perception of their position in life, encompassing physical, mental, emotional, and social functioning. | How a patient perceives their overall health and well-being in daily life. This includes the ability to perform daily activities, physical and mental health status, and the quality of social interactions. |
| Health outcomes patient | Measurable changes in disease-specific outcomes | Measurable indicators of a patient’s health status that reflect the effectiveness of a treatment/intervention. Examples include clinical metrics such as blood pressure, body weight, and blood glucose levels. |
| Quality of life informal caregiver | An individual’s perception of their position in life, encompassing physical, mental, emotional, and social functioning. | How the caregiver feels in their role and whether they are able to engage in activities they personally value. It includes emotional well-being, perceived strain, and the ability to maintain a fulfilling personal life while providing care. |
| ***Equity*** | | |
| Accessibility of care with limited health literacy | The degree to which individuals have the capacity to obtain, process, and understand basic health information and services needed to make appropriate health decisions. | The effort required to obtain healthcare services when facing challenges in understanding, processing, and applying health-related information. This includes difficulty scheduling (the appropriate) health care appointments. |
| Accessibility of care with low literacy | An individual’s ability to read, write, and speak in English and compute and solve problems at levels of proficiency necessary to function on the job and in society to achieve one’s goals, and to develop one’s knowledge and potential. | Disparities in access to health services due to limited literacy. This includes difficulties in understanding written communication, such as letters from health care providers, and challenges in completing (digital) forms. |
| Accessibility of care with limited digital skills | The ability to seek, find, understand, and appraise health information from electronic sources and apply the knowledge gained to addressing or solving a health problem. | Disparities in access to health services due to difficulties in using digital technologies. This includes challenges in communicating with healthcare providers through digital platforms, such as apps, and in submitting personal health data electronically. |
| Accessibility of care with limited physical abilities | Restrictions or lack of abilities in performance of the individual. | It's about patients with physical disabilities being able to receive care. For example, some buildings lack wheelchair access, and some digital devices are not suitable for patients with eye problems. |
| Accessibility of care with limited financial resources (financial strain) | Inability to meet financial needs and obligations. | The ability of patients to obtain needed health care when facing economic constraints. This includes financial barriers such as out-of-pocket costs, travel costs or co-pays that may discourage or prevent individuals from seeking care. |
| Accessibility of care due to poor accessibility of care location | Factors that hinder access to healthcare services. | The extent to which patients with mobility or sensory impairments can access health care services. Barriers may include physical inaccessibility of care facilities, such as buildings that are not wheelchair accessible, or digital tools that are not adapted for people with visual impairments. |
| Equity of care regardless of gender, culture or social group | The absence of avoidable or remediable differences among groups of people. | Health services are unfairly and partially provided to patients based on gender, cultural background, or social group. Barriers such as prejudice, language differences, and cultural misunderstandings resulting from different values, norms, or traditions can impede access to appropriate care and treatment. |
| ***Costs*** | | |
| Healthcare use | The use of healthcare to diagnose, cure, or ameliorate disease, to improve or maintain function, or to obtain information about their health status. | The extent and manner in which patients use health care services, including physician visits, hospitalizations, and medication use. |
| Healthcare costs | The actual costs of providing services related to the delivery of healthcare, including the costs of procedures, therapies, and medications. | The financial expenditures associated with the use of health care services. |
| Monitoring costs | Costs for digital health technology, infrastructure, and the time spent by medical professionals reviewing data. | The financial expenditures associated with remotely monitoring a patient’s health status. This may include costs associated with personnel, equipment, and monitoring infrastructure such as centralized monitoring centers. |
| Out-of-pocket costs | The amount of money a patient pays for indirect expenses that are not covered by a health insurance plan (e.g. stairlift, home adaptation). | Healthcare-related expenses paid directly by the patient. This may include the cost of medical devices (e.g., stair lifts), medications, or treatments that are not fully covered by insurance, as well as the cost of digital tools that the patient must finance themselves. |
| Travel costs patient | Costs related to the patient’s/caregiver’s travel. | The costs incurred by patients when travelling to receive health care services. This may include the cost of public transportation, fuel, or parking associated with hospital or clinic visits. |
| Productivity patient | Health-related productivity loss due to sick leave (absenteeism) and reduced performance while at work as a result of uncontrolled diseases or health risks (presenteeism). | A patient’s ability to perform paid or unpaid work. Disease and/or treatment may reduce a patient’s ability to work or affect his/her performance in occupational activities. |
| Productivity informal caregiver | The impact of caregiving in terms of economic losses or opportunity costs, also encompassing household and family management and training activities. | The extent to which the provision of care to a patient by an informal caregiver (family member/close acquaintance) affects the caregiver’s ability to engage effectively in paid or unpaid work. |
| Productivity healthcare provider | The ratio of work outputs to the resources (inputs) used to produce them. | The amount of time a healthcare provider spends on patient care. The adoption of digital technologies has the potential to change the duration and efficiency of the treatment process. |
| Costs outside of healthcare | Costs that are not directly related to the provision of healthcare services. | Indirect costs associated with disease that extend beyond the health care system and affect areas such as social welfare, education, and the environment. Such costs may include increased reliance on social services, reduced participation in education, or environmental impacts. |
| Costs health insurer | All the costs (potentially covered by insurance) made by organizations, people and their actions who primary intent is to promote, restore or maintain health. | The expenses incurred by the insurance company for the financing of health care services provided to the patient. |
| ***Provider experience*** | | |
| Provider satisfaction | An attitude or emotional response to one’s tasks as well as to the physical and social conditions of the workplace. | The degree to which healthcare providers are satisfied with their work and the impact they have on patients. It includes factors such as the sense of fulfillment derived from patient interactions and outcomes, the time required to provide care, satisfaction with the way care is delivered, the availability of assisting tools, and perceived workload. |
| Workload | Amount of work assigned to or expected from a worker in a specified time period. | How healthcare providers perceive their workload, which is influenced by amount of tasks and associated time pressure. Repetitive tasks may also contribute to mental and/or physical strain. |
| Quality of care | The ability of the clinician/provider to correctly diagnose or conduct an assessment/provide a treatment. | The provider’s ability to accurately assess and effectively treat a patient’s health status. It includes factors such as the provider’s confidence in the (digital) tools they and/or the patient are using. |
| Communication with patient | Mutual understanding between clinicians and patients regarding patients’ health needs, values, and perspective and sharing power and responsibility. | How healthcare providers perceive their communication with patients, including whether they feel their information is understood by the patient. |
| Patient involvement | The active effort of a healthcare provider to active patient participation in healthcare decision-making, treatment planning, and service development. | How well the provider is able to involve the patient in care and decision-making, for example in in jointly deciding on treatment. |
| Ease of use of technology for the healthcare provider | A person’s perception of the usability of a particular system or technology when used within a defined context. | How difficult it is for healthcare providers to use digital tools to treat a patient. They should be able to use the digital tools at the right time and in a way that is consistent with their work. In addition, digital tools need to work together logically. |
| Acceptance of technology | Behavioral intention to use the application which depends on the performance expectancy, effort expectancy and social influence. | The extent to which the healthcare provider is willing to use digital tools in their work. This may depend on the training and support they receive and whether their colleagues are using these tools. |
| ***Sustainability*** | | |
| Sustainability | The responsible interaction with the environmental to avoid depletion or degradation of natural resources and allow for long-term environmental quality. | The environmental impact of health care. For example, energy use, waste, and emissions from travel to medical appointments. |
| **Value aspects added after the first round to *Patient experience* domain** | | |
| Social environment | The relationships a person has with other individuals, the community, and the larger society. | How the patient’s social system (family, friends, informal caregivers) supports their care. This may include, for example, support with digital tools and emotional support related to their use. |
| Uncertainty due to self-measurements | Continuous monitoring can lead to higher patient anxiety, e.g. patients may become hyper-focused on every fluctuation in their readings. | The degree of uncertainty or stress a patient may experience from (regular) measurement of their own health values. This may be caused, for example, by uncertain results or fear of a bad result. |
| **Value aspects added after the first round by splitting up the value aspect *Sustainability*** | | |
| Energy use of technology | Direct electricity consumption required to operate hardware and software systems. | The amount of energy used by digital tools such as AI, apps, and sensors. This can have an impact on the environment, for example, by increasing/decreasing energy consumption in healthcare. |
| Sustainability of equipment | Sustainability of equipment is defined by the equipment’s repairability, modularity, and upgradability. | How long digital tools can be used and whether (parts of) digital tools can be reused. This can help reduce waste. |
| Travel pollution | Travelling for healthcare which is associated with an impact on the environment by lowering greenhouse gas emission and other air pollutants. | The impact of digital tools on how often patients need to travel for care, and what that means for the environment. For example, remote monitoring of health data may reduce the need for hospital visits. |
