# Supplementary material for: Developing a Core Outcome Set for the Evaluation of Remote Patient Monitoring Interventions Using the Sextuple Aim: Modified Delphi Study
Source: J Med Internet Res. 2026 Jul 15;28:e92863. doi: 10.2196/92863 (PMC13372298; doi:10.2196/92863)
Supplement: Multimedia Appendix 3 [file jmir-v28-e92863-s003.docx]

**Supplementary File 3 – Calculation for normalization results ranking aspects across domains**

To analyze the ranking within domains for each group, the mean was normalized so that it ranged between 0 and 100 for all aspects (regardless the number of aspects in each domain) using the formula:

$$normalized mean=100*\left( \frac{n-\min rank}{\max rank-\min rank} \right)$$

with *n* the mean ranking score, *max rank* is the highest ranking position within that domain and *min rank* is the lowest ranking position within that domain. The normalized mean was weighted by group size to obtain the results for the overall group.
