# Supplementary material for: Developing a Core Outcome Set for the Evaluation of Remote Patient Monitoring Interventions Using the Sextuple Aim: Modified Delphi Study
Source: J Med Internet Res. 2026 Jul 15;28:e92863. doi: 10.2196/92863 (PMC13372298; doi:10.2196/92863)
Supplement: Multimedia Appendix 4 [file jmir-v28-e92863-s004.docx]

**Supplementary File 4 – Informed consent Waiver: RPM Sextuple Aim COS – Delphi study**

**Informed Consent Waiver – Sextuple Aim COS**

Important: This questionnaire is completely anonymous. Your name and other personal information (with the exception of gender and age) will not be requested. Although the questionnaires will be sent to your email address, your response cannot be traced back to your email address.

1. I consent to the collection and use of my data for the purpose of answering the research question of this study. I also consent to the anonymous analysis and publication of the results.

□ Yes

1. I am 18 years of age or older, and I have a sufficient understanding of the Dutch language.

□ Yes

1. I consent to my data being retained for 15 years following this study.

□ Yes
