# Supplementary material for: Developing a Core Outcome Set for the Evaluation of Remote Patient Monitoring Interventions Using the Sextuple Aim: Modified Delphi Study
Source: J Med Internet Res. 2026 Jul 15;28:e92863. doi: 10.2196/92863 (PMC13372298; doi:10.2196/92863)
Supplement: Multimedia Appendix 5 [file jmir-v28-e92863-s005.docx]

**Supplementary File 5 – Figures value aspects without consensus across all groups (R1/R2/R3)**

**Figure 1 – Perceived importance of value aspects without consensus in the first Delphi round for all groups together and for each stakeholder group, presented as highest perceived importance (all groups) through lowest perceived importance (all groups)**

**Figure 2 – Perceived importance of value aspects without consensus in the second Delphi round for all groups together and for each stakeholder group, presented as highest perceived importance (all groups) through lowest perceived importance (all groups)**

**Figure 3 – Perceived importance of value aspects without consensus in the third Delphi round for all groups together and for each stakeholder group, presented as highest perceived importance (all groups) through lowest perceived importance (all groups)**
