# Supplementary material for: Developing a Core Outcome Set for the Evaluation of Remote Patient Monitoring Interventions Using the Sextuple Aim: Modified Delphi Study
Source: J Med Internet Res. 2026 Jul 15;28:e92863. doi: 10.2196/92863 (PMC13372298; doi:10.2196/92863)
Supplement: Multimedia Appendix 6 [file jmir-v28-e92863-s006.docx]

**Supplementary file 6 – Percentages of respondents who rated the importance of the value aspects in the last Delphi round they were presented with, per value aspect and stakeholder group**

| Value aspect | All groups | Patients | Inf careg | Provider | Manager | Insurer | Researcher | |
| --- | --- | --- | --- | --- | --- | --- | --- | --- |
| **Patient experience** | | | | | | | | |
| Patient satisfaction[1] | 99.4% | 100% | 97.8% | 100% | 100% | 100% | | 100% |
| Access to care[1] | 100% | 100% | 100% | 100% | 100% | 100% | | 100% |
| Information provision[1] | 100% | 100% | 100% | 100% | 100% | 100% | | 100% |
| Therapy adherence[1] | 100% | 100% | 100% | 100% | 100% | 100% | | 100% |
| Commun provider[1] | 100% | 100% | 100% | 100% | 100% | 100% | | 100% |
| Patient involvement[1] | 100% | 100% | 100% | 100% | 100% | 100% | | 100% |
| Perceived safety[1] | 99.4% | 100% | 97.8% | 100% | 100% | 100% | | 100% |
| Ease of use technology[1] | 100% | 100% | 100% | 100% | 100% | 100% | | 100% |
| Impact treatment[1] | 100% | 100% | 100% | 100% | 100% | 100% | | 100% |
| Self-control[1] | 100% | 100% | 100% | 100% | 100% | 100% | | 100% |
| Health knowledge[2] | 98.8%  100% | 98.3%  100% | 100%  100% | 100%  100% | 95.7%  100% | 100%  100% | | 100%  100% |
| Self-management[1] | 98.8% | 96.6% | 100% | 100% | 100% | 100% | | 100% |
| Technology adherence[1] | 98.8% | 96.6% | 100% | 100% | 100% | 100% | | 100% |
| Uncertainty measurement[3] | --  100%  95.6% | --  100%  100% | --  100%  97.3% | --  100%  100% | --  100%  100% | --  100%  75.0% | | --  100%  100% |
| Social system[3] | --  100%  98.5% | --  100%  98.1% | --  100%  97.3% | --  100%  100% | --  100%  100% | --  100%  100% | | --  100%  100% |
| Social contact[3] | 94.2%  100%  100% | 96.6%`  100%  100% | 95.6%  100%  100% | 100%  100%  100% | 95.7%  100%  100% | 100%  100%  100% | | 100%  100%  100% |
| Travel burden[3] | 98.2%  99.3%  99.3% | 96.6%  98.1%  100% | 100%  100%  97.3% | 100%  100%  100% | 95.7%  100%  100% | 100%  100%  100% | | 100%  100%  100% |
| **Health** | | | | | | | | |
| QoL patients[1] | 100% | 100% | 100% | 100% | 100% | 100% | | 100% |
| Health outcomes[1] | 99.4% | 96.6% | 100% | 100% | 100% | 100% | | 100% |
| QoL inf caregivers[1] | 94.2% | 86.2% | 97.8% | 100% | 91.3% | 100% | | 90.0% |
| **Equity** | | | | | | | | |
| Equality across groups[1] | 98.2% | 96.6% | 100% | 100% | 91.3% | 100% | | 100% |
| Limited physical abilities[1] | 98.8% | 98.3% | 100% | 100% | 95.7% | 100% | | 100% |
| Limited financial res[1] | 98.8% | 98.3% | 100% | 100% | 95.7% | 100% | | 100% |
| Limited literacy[1] | 100% | 100% | 100% | 100% | 100% | 100% | | 100% |
| Limited health literacy[1] | 99.4% | 98.3% | 97.8% | 100% | 100% | 100% | | 100% |
| Limited digital skills[1] | 99.4% | 100% | 97.8% | 100% | 100% | 100% | | 100% |
| Limited hc location[1] | 98.2% | 100% | 100% | 92.6% | 95.7% | 100% | | 100% |
| **Costs** | | | | | | | | |
| Healthcare costs[2] | 96.5%  100% | 93.1%  100% | 97.8%  100% | 100%  100% | 87.0%  100% | 100%  100% | | 100%  100% |
| Productivity provider[1] | 95.9% | 89.7% | 95.6% | 100% | 100% | 100% | | 90.0% |
| Healthcare use[1] | 95.3% | 94.8% | 95.6% | 96.3% | 95.7% | 100% | | 90.0% |
| Out-of-pocket costs[3] | 95.3%  98.6%  99.3% | 91.4%  98.1%  96.3% | 95.6%  100%  100% | 100%  94.4%  100% | 95.7%  100%  100% | 87.5%  100%  100% | | 100%  100%  100% |
| Costs health insurer[3] | 95.3%  99.3%  99.3% | 91.4%  100%  100% | 97.8%  100%  100% | 96.3%  94.4%  100% | 100%  100%  94.1% | 87.5%  100%  100% | | 100%  100%  100% |
| Productivity informal caregiver[3] | 93.0%  98.0%  98.5% | 87.9%  96.3%  91.4% | 86.7%  97.5%  100% | 96.3%  100%  100% | 95.7%  100%  100% | 100%  100%  100% | | 90.0%  100%  100% |
| Monitoring costs[3] | 96.5%  98.6%  99.3% | 100%  98.1%  96.3% | 100%  100%  100% | 96.3%  94.4%  100% | 87.0%  100%  100% | 100%  100%  100% | | 100%  100%  100% |
| Productivity patient[3] | 94.2%  98.6%  98.5% | 79.3%  98.1%  92.9% | 93.3%  97.5%  100% | 96.3%  100%  100% | 95.7%  100%  100% | 100%  100%  100% | | 100%  100%  100% |
| Travel costs[3] | 97.7%  98.6%  100% | 94.8%  100%  100% | 97.8%  97.5%  100% | 96.3%  94.4%  100% | 95.7%  100%  100% | 100%  100%  100% | | 100%  100%  100% |
| Costs outside healthcare[3] | 91.2%  98.0%  96.3% | 91.4%  98.1%  90.7% | 88.9%  97.5%  100% | 96.3%  94.4%  100% | 82.6%  100%  100% | 87.5%  100%  100% | | 100%  100%  90.0% |
| **Provider experience** | | | | | | | | |
| QoC[1] | 99.4% | 98.3% | 97.8% | 100% | 100% | 100% | | 100% |
| Patient involvement[1] | 99.4% | 100% | 97.8% | 100% | 100% | 100% | | 100% |
| Workload[1] | 96.5% | 91.4% | 97.8% | 100% | 100% | 100% | | 100% |
| Provider satisfaction[1] | 97.1% | 94.8% | 97.8% | 100% | 100% | 100% | | 100% |
| Ease of use technology[1] | 98.8% | 96.7% | 97.8% | 100% | 100% | 100% | | 100% |
| Communication patient[1] | 97.1% | 98.3% | 97.8% | 100% | 100% | 87.5% | | 100% |
| Acceptance technology[1] | 98.8% | 96.7% | 95.6% | 100% | 100% | 100% | | 100% |
| **Sustainability** | | | | | | | | |
| Sustainability[1] | 97.7% | 100% | 100% | 96.3% | 100% | 100% | | 90.0% |
| Reusability equipment[3] | --  99.3%  100% | --  98.1%  100% | --  100%  100% | --  100%  100% | --  100%  100% | --  100%  100% | | --  100%  100% |
| Pollution travel[3] | --  99.3%  100% | --  98.1%  100% | --  100%  100% | --  100%  100% | --  100%  100% | --  100%  100% | | --  100%  100% |
| Energy use[3] | --  100%  99.3% | --  100%  98.1% | --  100%  100% | --  100%  100% | --  100%  100% | --  100%  100% | | --  100%  100% |

[1] Round 1; [2] Round 2; [3] Round 3; Inf careg. = informal caregiver; Commun provider = communication with provider; Limited hc location = limited healthcare location; Qol = quality of life; QoC = quality of care
