# Supplementary material for: Developing a Core Outcome Set for the Evaluation of Remote Patient Monitoring Interventions Using the Sextuple Aim: Modified Delphi Study
Source: J Med Internet Res. 2026 Jul 15;28:e92863. doi: 10.2196/92863 (PMC13372298; doi:10.2196/92863)
Supplement: Multimedia Appendix 7 [file jmir-v28-e92863-s007.docx]

**Supplementary File 7 - Table Normalized mean ranking of value aspects within domains for all groups together and for each stakeholder group**

| Value aspect | All groups^1^ | Patients | Caregivers | Providers | Managers | Insurers | Researcher |
| --- | --- | --- | --- | --- | --- | --- | --- |
| ***Patient experience (mean, (SD))*** | | | | | | | |
| Self-control | 47.8 (38.3) | 43.4 (43.6) | 53.9 (42.4) | 52.4 (40.7) | 56.4 (36.1) | 42.9 (31.9) | 38.1 (37.1) |
| Access to care | 47.7 (40.8) | 28.6 (38.1) | 37.5 (41.1) | 48.4 (43.9) | 40.6 (39.9) | 62.9 (38.6) | 68.3 (36.3) |
| Impact treatment on life | 42.0 (40.3) | 38.9 (41.8) | 42.5 (40.9) | 24.6 (37.9) | 44.4 (42.0) | 57.1 (47.4) | 44.4 (33.1) |
| Self-management | 38.4 (36.1) | 31.7 (38.0) | 21.1 (30.0) | 50.8 (39.6) | 48.1 (38.2) | 48.6 (35.9) | 30.2 (30.7) |
| Patient satisfaction | 37.2 (38.0) | 23.0 (33.8) | 34.6 (40.0) | 23.8 (30.6) | 40.6 (42.1) | 45.7 (32.6) | 55.6 (43.7) |
| Therapy adherence | 28.1 (33.5) | 19.0 (28.2) | 33.6 (37.7) | 25.4 (31.6) | 32.3 (31.9) | 48.6 (44.7) | 9.5 (12.4) |
| Patient involvement | 26.5 (34.3) | 37.0 (36.2) | 28.6 (33.3) | 38.1 (39.2) | 28.6 (34.0) | 0.0 (0.0) | 27.0 (36.0) |
| Ease of use technology (patient) | 25.1 (28.6) | 31.0 (31.5) | 30.4 (30.9) | 24.6 (27.2) | 15.8 (22.3) | 31.4 (27.5) | 17.5 (32.6) |
| Communication with provider | 24.3 (32.5) | 43.4 (36.0) | 33.6 (33.1) | 10.3 (25.4) | 18.0 (26.9) | 8.6 (19.2) | 31.7 (39.6) |
| Perceived safety | 19.5 (31.2) | 17.2 (29.7) | 20.0 (31.8) | 32.5 (40.3) | 24.1 (31.6) | 2.9 (6.4) | 20.6 (32.8) |
| Information provision | 18.6 (28.5) | 23.5 (32.0) | 12.5 (24.5) | 26.2 (26.0) | 22.6 (32.4) | 2.9 (6.4) | 23.8 (36.4) |
| Health knowledge | 18.3 (27.5) | 23.0 (27.8) | 21.8 (30.9) | 29.4 (30.0) | 16.5 (29.1) | 0.0 (0.0) | 19.0 (27.7) |
| Social contact | 7.4 (21.7) | 6.9 (19.4) | 4.3 (10.3) | 3.2 (13.5) | 0.8 (3.3) | 22.9 (43.6) | 6.3 (19.0) |
| Travel burden | 5.4 (15.5) | 17.5 (28.5) | 9.6 (16.3) | 3.2 (9.2) | 2.3 (7.2) | 0.0 (0.0) | 0.0 (0.0) |
| Technology adherence | 4.9 (16.4) | 8.2 (18.9) | 3.2 (12.7) | 1.6 (4.6) | 2.3 (9.8) | 14.3 (31.9) | 0.0 (0.0) |
| Social system | 4.8 (13.7) | 5.6 (19.1) | 7.1 (16.8) | 4.0 (11.8) | 1.5 (4.5) | 5.7 (12.8) | 4.8 (14.3) |
| Measurement uncertainty | 3.9 (13.3) | 2.1 (7.6) | 5.7 (19.1) | 1.6 (6.7) | 5.3 (19.8) | 5.7 (12.8) | 3.2 (9.5) |
| ***Health (mean, (SD))*** | | | | | | | |
| QoL patient | 81.0 (29.6) | 83.3 (27.5) | 83.8 (32.8) | 91.7 (19.2) | 89.5 (20.9) | 60.0 (41.8) | 77.8 (26.4) |
| Health outcomes | 52.4 (27.8) | 47.2 (29.8) | 45.0 (25.0) | 50.0 (19.2) | 55.3 (15.8) | 50.0 (41.8) | 66.7 (16.7) |
| QoL informal caregiver | 16.6 (35.9) | 19.4 (36.9) | 21.3 (37.2) | 8.3 (29.7) | 5.3 (28.4) | 40.0 (50.0) | 5.6 (35.4) |
| ***Equity*** ***(mean, (SD))*** | | | | | | | |
| Limited health literacy | 64.0 (26.4) | 52.5 (27.7) | 53.3 (28.8) | 71.3 (30.1) | 60.5 (26.2) | 83.3 (11.8) | 63.0 (18.2) |
| Equality across groups | 60.6 (38.2) | 53.4 (39.2) | 55.4 (38.7) | 63.9 (38.9) | 32.5 (37.0) | 76.7 (32.5) | 81.5 (25.6) |
| Limited financial resources | 54.2 (29.1) | 58.6 (32.7) | 50.0 (30.0) | 49.1 (27.7) | 65.8 (28.6) | 36.7 (21.7) | 64.8 (26.9) |
| Limited physical abilities | 54.1 (31.6) | 62.3 (28.1) | 65.0 (27.4) | 56.5 (32.9) | 61.4 (34.7) | 50.0 (35.4) | 29.6 (21.7) |
| Limited digital skills | 42.9 (29.0) | 41.4 (30.0) | 43.3 (34.6) | 43.5 (26.9) | 56.1 (27.9) | 40.0 (19.0) | 33.3 (33.3) |
| Limited literacy | 42.9 (30.9) | 39.8 (33.2) | 32.5 (29.0) | 42.6 (28.1) | 38.6 (29.9) | 46.7 (32.1) | 57.4 (33.4) |
| Limited access healthcare location | 31.3 (35.1) | 42.0 (35.6) | 50.4 (36.5) | 23.1 (29.2) | 35.1 (34.6) | 16.7 (37.3) | 20.4 (30.9) |
| ***Costs (mean, (SD))*** | | | | | | | |
| Healthcare costs | 76.9 (28.3) | 59.8 (32.4) | 64.3 (35.4) | 81.7 (18.9) | 81.2 (25.7) | 88.6 (12.0) | 85.7 (28.6) |
| Healthcare use | 68.3 (31.4) | 55.6 (33.0) | 62.1 (35.8) | 69.0 (36.1) | 74.4 (25.9) | 77.1 (26.0) | 71.4 (30.3) |
| Productivity provider | 48.8 (33.9) | 46.0 (38.5) | 29.6 (30.9) | 54.8 (33.0) | 49.6 (35.4) | 71.4 (17.5) | 41.3 (33.8) |
| Out-of-pocket costs | 41.3 (34.0) | 57.9 (37.1) | 51.8 (34.3) | 39.7 (33.4) | 33.1 (30.5) | 11.4 (18.6) | 54.0 (27.5) |
| Patient productivity | 40.3 (32.9) | 39.9 (35.3) | 42.5 (35.9) | 56.3 (28.8) | 47.4 (32.3) | 14.3 (24.7) | 41.3 (28.1) |
| Monitoring costs | 32.8 (28.5) | 31.7 (29.6) | 32.9 (31.9) | 29.4 (31.2) | 42.1 (32.8) | 37.1 (16.3) | 23.8 (26.7) |
| Insurer costs | 27.5 (30.3) | 22.0 (27.6) | 21.1 (30.3) | 16.7 (22.6) | 24.8 (28.1) | 60.0 (23.5) | 20.6 (31.2) |
| Informal caregiver productivity | 27.3 (28.8) | 32.5 (33.7) | 30.7 (32.3) | 28.6 (25.0) | 24.1 (27.0) | 25.7 (35.6) | 22.2 (22.7) |
| Travel costs | 20.4 (27.3) | 38.1 (34.5) | 35.7 (32.5) | 12.7 (20.7) | 18.8 (20.2) | 2.9 (6.4) | 14.3 (22.6) |
| Costs outside healthcare | 16.4 (24.6) | 16.4 (23.8) | 29.3 (29.6) | 11.1 (18.0) | 4.5 (11.7) | 11.4 (15.6) | 25.4 (35.6) |
| ***Provider experience (mean, (SD))*** | | | | | | | |
| QoC | 86.3 (21.2) | 79.6 (27.2) | 82.9 (24.6) | 79.6 (23.3) | 88.6 (16.7) | 100.0 (0.0) | 87.0 (18.2) |
| Workload | 54.4 (30.2) | 42.9 (30.8) | 33.3 (25.0) | 54.6 (38.7) | 57.9 (26.3) | 63.3 (21.7) | 74.1 (20.6) |
| Patient involvement | 51.8 (29.6) | 53.7 (30.7) | 68.3 (26.4) | 46.3 (31.6) | 50.9 (29.1) | 40.0 (30.3) | 51.9 (28.2) |
| Communication with patient | 50.2 (32.0) | 57.1 (27.4) | 68.8 (25.4) | 54.6 (36.1) | 42.1 (31.6) | 43.3 (38.4) | 35.2 (26.9) |
| Provider satisfaction | 41.5 (30.4) | 35.8 (30.6) | 30.0 (30.5) | 41.7 (24.4) | 44.7 (35.6) | 46.7 (29.8) | 50.0 (32.3) |
| Ease of use technology (provider) | 34.5 (25.9) | 39.5 (30.2) | 34.6 (26.0) | 36.1 (30.4) | 36.0 (27.9) | 33.3 (26.4) | 27.8 (14.4) |
| Acceptance technology | 31.3 (31.0) | 41.4 (35.9) | 32.1 (26.8) | 37.0 (30.5) | 29.8 (31.2) | 23.3 (22.4) | 24.1 (38.3) |
| ***Sustainability (mean, (SD))*** | | | | | | | |
| Reusability equipment | 73.3 (35.2) | 74.1 (31.8) | 70.0 (35.4) | 75.0 (30.9) | 68.4 (29.9) | 80.0 (44.7) | 72.2 (44.1) |
| Pollution travel | 42.7 (41.3) | 48.2 (42.3) | 45.0 (45.0) | 38.9 (47.1) | 55.3 (43.8) | 30.0 (27.4) | 38.9 (41.7) |
| Energy use equipment | 34.0 (35.1) | 27.8 (34.6) | 35.0 (34.3) | 36.1 (33.5) | 26.3 (38.6) | 40.0 (41.8) | 38.9 (33.3) |

Green = value aspect mean rank above the domain average; orange = value aspect mean rank below the domain average. ^1^results were weighted to account for differences in group sizes. SD = standard deviation; QoL = Quality of Life; QoC = Quality of Care.
