# Supplementary material for: Developing a Core Outcome Set for the Evaluation of Remote Patient Monitoring Interventions Using the Sextuple Aim: Modified Delphi Study
Source: J Med Internet Res. 2026 Jul 15;28:e92863. doi: 10.2196/92863 (PMC13372298; doi:10.2196/92863)
Supplement: Multimedia Appendix 8 [file jmir-v28-e92863-s008.docx]

**Supplementary File 8 – Table with ranking score of the importance of value aspects across all domains for all groups together and for each stakeholder group**

| ***Value aspect*** | *All groups^1^* | *Patients* | | *Inf. Caregivers* | | *Providers* | | *Managers* | | *Insurers* | | *Researchers* | |
| --- | --- | --- | --- | --- | --- | --- | --- | --- | --- | --- | --- | --- | --- |
|  | Mean (SD) | Mean (SD) | P | Mean (SD) | P | Mean (SD) | P | Mean (SD) | P | Mean (SD) | P | Mean (SD) | P |
| 1. QoL patient | 5.61 (4.09) | 4.41 (41.6) | 1 | 5.43 (4.30) | 1 | 6.40 (4.36) | 1 | 4.41 (4.37) | 3 | 7.75 (2.06) | 1 | 5.25 (4.46) | 1 |
| 2. QoC | 3.67 (3.95) | 2.38 (3.62) | 7 | 2.92 (3.79) | 4 | 3.00 (3.93) | 5 | 5.35 (4.23) | 1 | 5.00 (3.56) | 4 | 3.38 (4.57) | 5 |
| 3. Access to care | 3.33 (3.97) | 1.21 (2.30) |  | 2.08 (3.29) | 9 | 1.33 (3.52) |  | 4.71 (4.28) | 2 | 6.50 (4.73) | 3 | 4.13 (3.40) | 3 |
| 4. Health outcomes | 3.21 (4.27) | 1.00 (2.74) |  | 0.70 (2.37) |  | 3.27 (4.23) | 4 | 3.18 (4.52) | 7 | 7.00 (4.69) | 2 | 4.13 (4.42) | 2 |
| 5. Self-control | 3.01 (3.69) | 4.07 (4.09) | 2 | 4.00 (3.84) | 2 | 3.60 (3.87) | 3 | 4.00 (3.67) | 5 | 0.25 (0.50) |  | 2.13 (3.64) | 10 |
| 6. Self-management | 2.83 (3.51) | 2.43 (3.60) | 6 | 1.86 (3.03) | 10 | 3.60 (3.60) | 2 | 4.06 (3.61) | 4 | 1.25 (2.50) |  | 3.75 (4.30) | 4 |
| 7. Healthcare costs | 2.39 (3.10) | 1.32 (2.94) |  | 1.03 (2.37) |  | 2.33 (3.04) | 7 | 2.65 (3.44) | 9 | 4.25 (3.10) | 5 | 2.75 (3.33) | 6 |
| 8. Patient satisfaction | 2.17 (3.03) | 1.46 (2.67) |  | 2.35 (3.09) | 7 | 2.67 (3.68) | 6 | 1.65 (3.08) |  | 4.00 (2.71) | 6 | 0.88 (2.47) |  |
| 9. Impact treatment on life | 2.03 (3.24) | 2.21 (3.55) | 8 | 3.65 (4.32) | 3 | 1.00 (2.10) |  | 0.82 (2.43) |  | 2.25 (3.86) |  | 2.25 (2.55) |  |
| 10. Healthcare use | 1.72 (2.95) | 0.84 (2.06) |  | 0.22 (1.16) |  | 1.33 (2.44) |  | 3.29 (3.58) | 6 | 2.25 (2.63) | 10 | 2.38 (4.41) | 9 |
| 11. Patient involvement | 1.68 (3.03) | 1.39 (2.77) |  | 2.49 (3.84) | 6 | 1.40 (2.59) |  | 3.06 (3.36) | 8 | 0.00 (0.00) |  | 1.75 (3.41) |  |
| 12. Workload | 1.56 (2.54) | 0.41 (1.70) |  | 0.30 (1.05) |  | 1.47 (3.31) |  | 1.59 (2.53) |  | 3.00 (2.71) | 7 | 2.63 (2.72) | 7 |
| 13. Communication with patient | 1.51 (3.11) | 2.77 (3.63) | 4 | 2.11 (3.60) | 8 | 0.00 (0.00) |  | 0.53 (2.18) |  | 2.25 (4.50) | 9 | 1.38 (2.77) |  |
| 14. Communication with provider | 1.38 (2.71) | 3.14 (3.67) | 3 | 2.86 (3.63) | 5 | 0.47 (1.06) |  | 0.53 (1.50) |  | 0.00 (0.00) |  | 1.25 (2.55) |  |
| 15. Equality across groups | 1.23 (2.86) | 1.50 (2.61) |  | 1.68 (2.98) |  | 1.53 (3.23) |  | 0.18 (0.53) |  | 0.00 (0.00) |  | 2.50 (4.63) | 8 |
| 16. Productivity provider | 1.15 (2.35) | 0.70 (2.17) |  | 0.43 (1.37) |  | 0.67 (1.80) |  | 0.88 (1.87) |  | 2.75 (3.40) | 8 | 1.50 (2.98) |  |
| 17. Limited digital skills | 1.06 (2.19) | 0.88 (2.16) |  | 1.00 (1.96) |  | 1.60 (2.90) | 10 | 0.65 (1.54) |  | 0.75 (1.50) |  | 1.50 (2.98) |  |
| 18. Therapy adherence | 1.06 (2.44) | 0.80 (1.92) |  | 1.43 (2.67) |  | 1.60 (2.92) |  | 0.65 (2.03) |  | 1.00 (2.00) |  | 0.88 (1.64) |  |
| 19. Ease of techn. patient | 1.03 (2.44) | 2.43 (3.36) | 5 | 1.73 (3.13) |  | 1.33 (3.02) |  | 0.59 (1.33) |  | 0.00 (0.00) |  | 0.13 (0.35) |  |
| 20. Limited health literacy | 1.01 (2.16) | 1.07 (2.38) |  | 0.62 (1.67) |  | 1.13 (2.47) |  | 1.24 (2.39) |  | 0.50 (1.00) |  | 1.50 (2.83) |  |
| 21. Perceived safety | 0.96 (2.32) | 1.05 (2.31) |  | 0.46 (1.95) |  | 2.13 (3.60) | 8 | 0.76 (2.05) |  | 0.00 (0.00) |  | 1.38 (2.20) |  |
| 22. Involvement patient (prov.e) | 0.92 (2.33) | 1.82 (3.09) | 9 | 0.97 (2.11) |  | 1.67 (3.18) | 9 | 1.06 (2.54) |  | 0.00 (0.00) |  | 0.00 (0.00) |  |
| 23. Provider satisfaction | 0.89 (2.17) | 0.41 (1.58) |  | 0.81 (2.12) |  | 0.87 (2.36) |  | 1.76 (2.68) | 10 | 0.00 (0.00) |  | 1.50 (2.83) |  |
| 24. QoL informal caregiver | 0.86 (2.23) | 0.86 (2.56) |  | 1.19 (2.40) |  | 0.60 (2.06) |  | 0.53 (2.18) |  | 1.25 (2.50) |  | 0.75 (2.12) |  |
| 25. Limited financial resources | 0.79 (2.21) | 1.02 (2.35) |  | 0.73 (2.12) |  | 1.00 (2.20) |  | 0.00 (0.00) |  | 2.00 (4.00) |  | 0.00 (0.00) |  |
| 26. Information provision | 0.72 (1.86) | 0.95 (2.07) |  | 1.41 (2.94) |  | 1.07 (1.71) |  | 0.88 (1.96) |  | 0.00 (0.00) |  | 0.00 (0.00) |  |
| 27. Limited physical abilities | 0.68 (2.05) | 1.13 (2.70) |  | 1.54 (3.24) |  | 0.87 (1.96) |  | 0.53 (1.50) |  | 0.00 (0.00) |  | 0.00 (0.00) |  |
| 28. Limited literacy | 0.65 (1.92) | 0.23 (0.89) |  | 0.65 (2.07) |  | 1.40 (2.97) |  | 0.65 (1.90) |  | 0.00 (0.00) |  | 1.00 (2.14) |  |
| 29. Out-of-pocket costs | 0.62 (1.76) | 1.55 (2.78) | 10 | 1.19 (2.26) |  | 0.33 (1.05) |  | 0.00 (0.00) |  | 0.00 (0.00) |  | 0.63 (1.77) |  |
| 30. Health knowledge | 0.58 (1.71) | 1.09 (2.44) |  | 0.76 (1.64) |  | 1.00 (2.20) |  | 0.65 (1.84) |  | 0.00 (0.00) |  | 0.00 (0.00) |  |
| 31. Productivity patient | 0.56 (1.63) | 0.57 (1.86) |  | 0.73 (1.91) |  | 1.40 (2.23) |  | 0.65 (1.73) |  | 0.00 (0.00) |  | 0.00 (0.00) |  |
| 32. Ease of use techn. provider | 0.54 (1.62) | 0.70 (2.07) |  | 0.59 (1.64) |  | 0.53 (1.05) |  | 1.18 (2.38) |  | 0.00 (0.00) |  | 0.25 (0.71) |  |
| 33. Travel burden | 0.44 (1.38) | 1.34 (2.44) |  | 0.78 (1.62) |  | 0.53 (1.25) |  | 0.00 (0.00) |  | 0.00 (0.00) |  | 0.00 (0.00) |  |
| 34. Health insurers cost | 0.38 (1.20) | 0.25 (1.05) |  | 0.32 (1.13) |  | 0.20 (0.56) |  | 0.53 (1.94) |  | 0.75 (1.50) |  | 0.25 (0.71) |  |
| 35. Social contact | 0.37 (1.36) | 0.95 (2.14) |  | 0.27 (1.64) |  | 0.27 (1.03) |  | 0.24 (0.75) |  | 0.00 (0.00) |  | 0.50 (1.41) |  |
| 36. Travel costs | 0.36 (1.43) | 1.38 (2.91) |  | 0.59 (1.50) |  | 0.07 (0.26) |  | 0.00 (0.00) |  | 0.00 (0.00) |  | 0.13 (0.35) |  |
| 37. Reusability equipment | 0.36 (1.09) | 0.70 (2.04) |  | 0.30 (0.81) |  | 0.60 (1.18) |  | 0.06 (0.24) |  | 0.25 (0.50) |  | 0.25 (0.71) |  |
| 38. Limited access healthcare loc. | 0.35 (1.54) | 0.50 (1.56) |  | 0.46 (1.41) |  | 0.00 (0.00) |  | 0.00 (0.00) |  | 0.00 (0.00) |  | 1.13 (3.18) |  |
| 39. Social system | 0.30 (1.36) | 0.79 (2.18) |  | 0.49 (1.37) |  | 0.53 (2.07) |  | 0.00 (0.00) |  | 0.00 (0.00) |  | 0.00 (0.00) |  |
| 40. Monitoring costs | 0.20 (0.81) | 0.07 (0.42) |  | 0.16 (0.69) |  | 0.20 (0.77) |  | 0.65 (1.58) |  | 0.00 (0.00) |  | 0.13 (0.35) |  |
| 41. Pollution travel | 0.18 (0.98) | 0.25 (1.03) |  | 0.62 (2.09) |  | 0.00 (0.00) |  | 0.06 (0.24) |  | 0.00 (0.00) |  | 0.13 (0.35) |  |
| 42. Productivity informal caregiver | 0.17 (0.97) | 0.30 (1.44) |  | 0.43 (1.44) |  | 0.00 (0.00) |  | 0.29 (1.21) |  | 0.00 (0.00) |  | 0.00 (0.00) |  |
| 43. Uncertainty measurements | 0.15 (0.72) | 0.16 (0.89) |  | 0.11 (0.46) |  | 0.00 (0.00) |  | 0.12 (0.49) |  | 0.00 (0.00) |  | 0.50 (1.41) |  |
| 44. Acceptance technology | 0.14 (0.73) | 0.07 (0.48) |  | 0.03 (0.16) |  | 0.00 (0.00) |  | 0.24 (0.97) |  | 0.00 (0.00) |  | 0.50 (1.41) |  |
| 45. Energy use | 0.07 (0.53) | 0.23 (0.66) |  | 0.03 (0.16) |  | 0.00 (0.00) |  | 0.18 (0.73) |  | 0.00 (0.00) |  | 0.00 (0.00) |  |
| 46. Costs outside healthcare | 0.06 (0.46) | 0.09 |  | 0.30 (1.00) |  | 0.00 (0.00) |  | 0.00 (0.00) |  | 0.00 (0.00) |  | 0.00 (0.00) |  |
| 47. Technology adherence | 0.05 (0.45) | 0.13 |  | 0.19 (0.88) |  | 0.00 (0.00) |  | 0.00 (0.00) |  | 0.00 (0.00) |  | 0.00 (0.00) |  |

^1^ Results were weighted to account for differences in group sizes; P position of the value aspect in the group ranking. prov.e = provider experience; First, participants selected 10 value aspects and rated them from most important (10) to least important (1).
