# Supplementary material for: Developing a Core Outcome Set for the Evaluation of Remote Patient Monitoring Interventions Using the Sextuple Aim: Modified Delphi Study
Source: J Med Internet Res. 2026 Jul 15;28:e92863. doi: 10.2196/92863 (PMC13372298; doi:10.2196/92863)
Supplement: Multimedia Appendix 9 [file jmir-v28-e92863-s009.docx]

**Supplementary File 9 – Sensitivity analysis mean ranking in case of deprioritization instead of neutrality unselected value aspects**

| Value aspect | Weighted mean ranking, 0 (SD) | Weighted mean ranking, -10 (SD) | Difference position |
| --- | --- | --- | --- |
| 1. QoL patient | 5.61 (4.09) | 2.58 (8.49) | 1 |
| 2. QoC | 3.67 (3.95) | -0.98 (8.66) | 2 |
| 3. Access to care | 3.33 (3.97) | -1.60 (8.60) | 3 |
| 4. Health outcomes | 3.21 (4.27) | -3.03 (9.07) | 6 |
| 5. Self-control | 3.01 (3.69) | -1.94 (8.29) | 4 |
| 6. Self-management | 2.83 (3.51) | -2.58 (8.25) | 5 |
| 7. Healthcare costs | 2.39 (3.10) | -3.22 (7.84) | 7 |
| 8. Patient satisfaction | 2.17 (3.03) | -3.63 (7.68) | 8 |
| 9. Impact treatment on life | 2.03 (3.24) | -4.52 (7.75) | 10 |
| 10. Healthcare use | 1.72 (2.95) | -5.19 (7.36) | 11 |
| 11. Patient involvement | 1.68 (3.03) | -5.43 (7.34) | 12 |
| 12. Workload | 1.56 (2.54) | -4.44 (7.04) | 9 |
| 13. Communication with patient | 1.51 (3.11) | -6.15 (7.14) | 14 |
| 14. Communication with provider | 1.38 (2.71) | -6.03 (6.87) | 13 |
| 15. Equality across groups | 1.23 (2.86) | -6.57 (6.69) | 16 |
| 16. Productivity provider | 1.15 (2.35) | -6.62 (6.41) | 17 |
| 17. Limited digital skills | 1.06 (2.19) | -6.45 (6.30) | 15 |
| 18. Therapy adherence | 1.06 (2.44) | -6.69 (6.24) | 19 |
| 19. Ease of technology patient | 1.03 (2.44) | -6.68 (6.31) | 18 |
| 20. Limited health literacy | 1.01 (2.16) | -6.85 (6.13) | 20 |
| 21. Perceived safety | 0.96 (2.32) | -7.14 (6.05) | 21 |
| 22. Involvement patient (prov.e) | 0.92 (2.33) | -7.52 (5.86) | 23 |
| 23. Provider satisfaction | 0.89 (2.17) | -7.46 (5.79) | 22 |
| 24. QoL informal caregiver | 0.86 (2.23) | -7.61 (5.72) | 25 |
| 25. Limited financial resources | 0.79 (2.21) | -7.88 (5.51) | 27 |
| 26. Information provision | 0.72 (1.86) | -7.54 (5.47) | 24 |
| 27. Limited physical abilities | 0.68 (2.05) | -8.04 (5.23) | 30 |
| 28. Limited literacy | 0.65 (1.92) | -8.10 (5.12) | 31 |
| 29. Out-of-pocket costs | 0.62 (1.76) | -7.97 (5.10) | 29 |
| 30. Health knowledge | 0.58 (1.71) | -8.22 (4.89) | 32 |
| 31. Productivity patient | 0.56 (1.63) | -7.87 (5.05) | 26 |
| 32. Ease of use technology provider | 0.54 (1.62) | -8.23 (4.80) | 33 |
| 33. Travel burden | 0.44 (1.38) | -8.28 (4.57) | 35 |
| 34. Health insurers cost | 0.38 (1.20) | -8.24 (4.48) | 34 |
| 35. Social contact | 0.37 (1.36) | -8.74 (4.10) | 37 |
| 36. Travel costs | 0.36 (1.43) | -8.60 (4.23) | 36 |
| 37. Reusability equipment | 0.36 (1.09) | -7.95 (4.62) | 28 |
| 38. Limited access healthcare loc | 0.35 (1.54) | -8.98 (3.90) | 39 |
| 39. Social system | 0.30 (1.36) | -9.12 (3.61) | 40 |
| 40. Monitoring costs | 0.20 (0.81) | -8.89 (3.56) | 38 |
| 41. Pollution travel | 0.18 (0.98) | -9.13 (3.28) | 41 |
| 42. Productivity informal caregiver | 0.17 (0.97) | -9.43 (2.85) | 43 |
| 43. Uncertainty measurements | 0.15 (0.72) | -9.40 (2.78) | 42 |
| 44. Acceptance technology | 0.14 (0.73) | -9.48 (2.63) | 44 |
| 45. Energy use | 0.07 (0.53) | -9.67 (2.07) | 45 |
| 46. Costs outside healthcare | 0.06 (0.46) | -9.70 (1.96) | 46 |
| 47. Technology adherence | 0.05 (0.45) | -9.80 (1.65) | 47 |

SD = standard deviation; 0 = neutrality; -10 = deprioritization; QoL = quality of life; QoC = quality of care; prov.e = provider experience; Limited access healthcare loc = limited access to healthcare location
