# Supplementary material for: Developing a Core Outcome Set for the Evaluation of Remote Patient Monitoring Interventions Using the Sextuple Aim: Modified Delphi Study
Source: J Med Internet Res. 2026 Jul 15;28:e92863. doi: 10.2196/92863 (PMC13372298; doi:10.2196/92863)
Supplement: Multimedia Appendix 10 [file jmir-v28-e92863-s010.docx]

**Supplementary File 10 – Table Preliminary Core Outcome Set based on multicriteria consensus rule**

| **Value aspect** | **Rating (R1/R2/R3), % importance (IQR)** | **Ranking within domain (R2), normalized mean** | **Ranking across domains (R3), mean (P)** |
| --- | --- | --- | --- |
| **Patient experience** | | | |
| Self-control | **85.8 (1)** | **47.8** | **3.01 (5)** |
| Access to care | **93.4 (1)** | **47.7** | **3.33 (3)** |
| Impact treatment on life | **88.4 (1)** | **42.0** | **2.03 (9)** |
| Self-management | **83.4 (1)** | **38.4** | **2.83 (6)** |
| Patient satisfaction | **94.6 (1)** | **37.2** | **2.17 (8)** |
| Therapy adherence | **92.0 (1)** | **28.1** | **1.06 (18)** |
| Patient involvement | **90.2 (1)** | **26.5** | **1.68 (11)** |
| Ease of use technology | **88.7 (1)** | **25.1** | **1.03 (19)** |
| Communication with provider | **91.8 (1)** | **24.3** | **1.38 (14)** |
| Perceived safety | **89.5 (1)** | 17.2 | **0.96 (21)** |
| **Health** | | | |
| QoL patient | **97.7 (1)** | **83.3** | **5.61 (1)** |
| Health outcomes | **93.3 (1)** | **47.2** | **3.21 (4)** |
| QoL informal caregiver | **78.5 (1)** | 19.4 | **0.86 (24)** |
| **Equity** | | | |
| Limited health literacy | **90.7 (1)** | **64.0** | **1.01 (20)** |
| Equality across groups | **95.4 (1)** | **60.6** | **1.23 (15)** |
| Limited financial resources | **92.5 (1)** | **54.2** | **0.79 (25)** |
| Limited physical abilities | **95.3 (1)** | **54.1** | 0.68 (27) |
| Limited digital skills | **89.8 (1)** | 42.9 | **1.06 (17)** |
| **Costs** |  |  |  |
| Healthcare costs | **86.1 (1)** | **76.9** | **2.39 (7)** |
| Healthcare use | **76.6 (1)** | **68.3** | **1.72 (10)** |
| Provider productivity | **82.5 (1)** | **48.8** | **1.15 (16)** |
| **Provider experience** | | | |
| QoC | **98.7 (0)** | **86.3** | **3.67 (2)** |
| Workload | **87.8 (1)** | **54.4** | **1.56 (12)** |
| Patient involvement | **89.9 (1)** | **51.8** | **0.92 (22)** |
| Communication with patient | **85.7 (1)** | **50.2** | **1.51 (11)** |
| Provider satisfaction | **86.3 (1)** | 41.5 | **0.89 (23)** |
| **Sustainability** | | | |
| -- | -- | -- | -- |

**R1 = Round 1; R2 = Round 2; R3 = Round 3; IQR = interquartile range; P = position; QoL = quality of life; QoC = quality of care**
