# Supplementary material for: Developing a Core Outcome Set for the Evaluation of Remote Patient Monitoring Interventions Using the Sextuple Aim: Modified Delphi Study
Source: J Med Internet Res. 2026 Jul 15;28:e92863. doi: 10.2196/92863 (PMC13372298; doi:10.2196/92863)
Supplement: Multimedia Appendix 11 [file jmir-v28-e92863-s011.docx]

**Supplementary File 11 – Complete case analysis importance-rating and importance-ranking across domains**

| **Value aspect [final round rated]** | Final percentage perceived importance (IQR) |
| --- | --- |
| **Patient experience** | |
| Patient satisfaction[1] | 93.1 (1.0) |
| Access to care[1] | 90.5 (1.0) |
| Information provision[1] | 97.7 (1.0) |
| Therapy adherence[1] | 93.2 (1.0) |
| Communication provider[1] | 94.0 (1.0) |
| Patient involvement[1] | 93.5 (1.0) |
| Perceived safety[1] | 86.5 (1.0) |
| Ease of use technology[1] | 90.9 (1.0) |
| Impact treatment[1] | 86.3 (1.0) |
| Self-control[1] | 83.3 (1.0) |
| Health knowledge[2] | 84.3 (1.0) |
| Self-management[1] | 81.6 (1.0) |
| Technology adherence[1] | 74.2 (1.0) |
| Uncertainty measurements[3] | 73.4 (1.0) |
| Social system[3] | 73.0 (2.0) |
| Social contact[3] | 58.7 (2.0) |
| Travel burden[3] | 35.3 (2.0) |
| **Health** |  |
| QoL patients[1] | 97.2 (1.0) |
| Health outcomes[1] | 92.1 (1.0) |
| QoL informal caregiver[1] | 75.8 (1.0) |
| **Equity** |  |
| Equality across groups[1] | 96.3 (1.0) |
| Limited physical abilities[1] | 96.9 (1.0) |
| Limited financial resources[1] | 92.8 (1.0) |
| Limited literacy[1] | 88.7 (1.0) |
| Limited health literacy[1] | 91.2 (1.0) |
| Limited digital skills[1] | 91.3 (1.0) |
| Limited healthcare location[1] | 73.0 (2.0) |
| **Costs** |  |
| Healthcare costs[2] | 85.1 (1.0) |
| Productivity provider[1] | 85.5 (1.0) |
| Healthcare utilization[1] | 75.6 (1.0) |
| Out-of-pocket costs[3] | 69.5 (2.0) |
| Costs health insurer[3] | 59.7 (1.0) |
| Productivity informal caregiver[3] | 59.4 (1.0) |
| Monitoring costs[3] | 58.7 (1.0) |
| Productivity patient[3] | 56.8 (1.0) |
| Travel costs[3] | 40.5 (2.0) |
| Costs outside of healthcare[3] | 39.6 (2.0) |
| **Provider experience** |  |
| QoC[1] | 98.2 (1.0) |
| Patient involvement[1] | 90.7 (1.0) |
| Workload[1] | 92.9 (1.0) |
| Provider satisfaction[1] | 84.9 (1.0) |
| Ease of use technology[1] | 87.0 (1.0) |
| Communication patient[1] | 80.9 (1.0) |
| Acceptance technology[1] | 82.2 (1.0) |
| **Sustainability** |  |
| *Sustainability[1]* | 53.6 (1.0) |
| Reusability equipment[3] | 57.0 (1.0) |
| Pollution travel[3] | 30.8 (2.0) |
| Energy use[3] | 13.7 (1.0) |

Table – Complete-case analysis importance rating (R1, R2, and R3)

^1^Results were weighted to correct for differences in sample size

≥ 0 - < 12.5; ≥ 12.5 - < 25.0; ≥ 25.0 - < 37.5; ≥ 37.5 - < 50.0; ≥ 50.0- < 62.5; ≥ 62.5- < 75.0; ≥ 75.0- < 87.5; ≥ 87.5 – 100;

QoL = quality of life; QoC = quality of care; IQR = interquartile range; [Final round rated] = the last round in which a value aspect was rated, when consensus was reached it was not rerated in the subsequent round. Final percentage perceived

Table – Complete-case analysis normalized mean ranking of value aspects within groups

| Value aspect | Normalized mean ranking (SD) |
| --- | --- |
| **Patient experience** | |
| Self-control | 48.6 (37.5) |
| Access to care | 46.4 (39.6) |
| Impact treatment on life | 44.2 (40.3) |
| Self-management | 37.4 (36.5) |
| Patient satisfaction | 39.6 (37.9) |
| Therapy adherence | 27.1 (33.1) |
| Patient involvement | 28.2 (35.5) |
| Ease of use technology (patient) | 23.3 (26.9) |
| Communication with provider | 24.6 (33.0) |
| Perceived safety | 20.1 (31.5) |
| Information provision | 16.3 (26.5) |
| Health knowledge | 18.6 (26.5) |
| Social contact | 6.9 (22.9) |
| Travel burden | 5.6 (15.6) |
| Technology adherence | 5.9 (17.8) |
| Social system | 2.9 (11.6) |
| Measurement uncertainty | 4.2 (14.0) |
| **Health** | |
| QoL patient | 79.3 (30.6) |
| Health outcomes | 53.2 (36.4) |
| QoL informal caregiver | 17.6 (29.0) |
| **Equity** | |
| Limited health literacy | 64.6 (26.1) |
| Equality across groups | 58.8 (39.0) |
| Limited financial resources | 55.1 (28.1) |
| Limited physical abilities | 54.6 (32.6) |
| Limited digital skills | 42.1 (28.5) |
| Limited literacy | 43.9 (31.6) |
| Limited access healthcare location | 31.0 (34.3) |
| **Costs** | |
| Healthcare costs | 75.9 (29.0) |
| Healthcare use | 67.0 (32.3) |
| Productivity provider | 49.4 (34.0) |
| Out-of-pocket costs | **37.2 (34.0)** |
| Patient productivity | 42.6 (33.4) |
| Monitoring costs | 34.4 (28.1) |
| Insurer costs | 27.1 (30.3) |
| Informal caregiver productivity | 30.9 (29.4) |
| Travel costs | 19.9 (28.3) |
| Costs outside healthcare | 15.6 (23.6) |
| **Provider experience** | |
| QoC | 86.4 (21.5) |
| Workload | 56.4 (29.0) |
| Patient involvement | 50.9 (30.3) |
| Communication with patient | 50.2 (31.3) |
| Provider satisfaction | 40.1 (29.7) |
| Ease of use technology (provider) | 36.0 (25.9) |
| Acceptance technology | 30.0 (31.7) |
| **Sustainability** | |
| Reusability equipment | 73.3 (35.0) |
| Pollution travel | 42.2 (42.4) |
| Energy use equipment | 34.5 (34.1) |

Green = value aspect mean rank above the domain average; orange = value aspect mean rank below the domain average. ^1^results were weighted to account for differences in group sizes. SD = standard deviation; QoL = Quality of Life; QoC = Quality of Care
